# AATF supports proliferation of glioblastoma cells by sustaining mitochondrial respiration through an NRF-1-dependent mechanism

**DOI:** 10.1038/s41419-026-08617-0

**Published:** 2026-03-24

**Authors:** Cristina Sorino, Stefano Di Giovenale, Italia Falcone, Francesca Romana Auciello, Claudio Pulito, Federica Lo Sardo, Stefano Scalera, Francesca De Nicola, Valeria Catena, Ludovica Ciuffreda, Brindusa Ana Maria Arteni, Stefano Giuliani, Bruno Amadio, Giovanni Blandino, Maurizio Fanciulli, Simona Iezzi

**Affiliations:** 1 Gene Expression and Cancer Models Unit, Department of Research and Advanced Technologies, Translational Research Area, IRCCS Regina Elena National Cancer Institute, Rome, Italy; 2 Department of Computer, Control and Management Engineering, Sapienza University of Rome, Rome, Italy; 3 Translational Oncology Research Unit, IRCCS Regina Elena National Cancer Institute, Rome, Italy; 4 Biostatistics, Bioinformatics and Clinical Trial Center, IRCCS Regina Elena National Cancer Institute, Rome, Italy; 5 Pathology Unit, Tissue Biobank, IRCCS Regina Elena National Cancer Institute, Rome, Italy

**Keywords:** Cancer, Cancer metabolism

## Abstract

The ability of cancer cells to promote cellular proliferation by preferentially using glycolysis as primary source of energy has long been considered a hallmark of tumour metabolism. However, emerging evidence suggests a more complex situation with many tumours exhibiting a pronounced dependence on mitochondrial respiration through oxidative phosphorylation (OXPHOS) for their development and maintenance. In line with this, numerous studies have reported an upregulation of mitochondrial genes and OXPHOS components across multiple cancer types. Glioblastoma (GBM) is the most frequent and malignant brain tumour in adults, characterised by rapid proliferation, resistance to therapy and ability to recur. In addition to a profound genetic and molecular heterogeneity, GBM also exhibits strong metabolic heterogeneity with different grades of dependence on mitochondrial activity. Notably, the transcription factor Nuclear Respiratory Factor 1 (NRF-1), a key regulator of OXPHOS gene expression and mitochondrial functions, has recently been linked to GBM progression and poor prognosis. Che-1/Apoptosis Antagonising Transcription Factor (AATF) is a transcriptional regulator with a crucial role in several cancer types, where it contributes to tumorigenesis by promoting cell cycle arrest and apoptosis, as well as resistance to therapy. Here, we show that AATF expression correlates with clinical outcome in GBM patients. Moreover, we demonstrate that its depletion leads to cell cycle arrest, impaired mitochondrial respiration and disrupted mitochondrial architecture in GBM cells. Additionally, AATF-depleted cells exhibit a reduced ability to form colonies in vitro and tumour in vivo. At the molecular level, we provide evidence that AATF interacts with NRF-1 and is essential for NRF-1-mediated transcription of the OXPHOS genes by affecting RNA polymerase II recruitment and chromatin structure. Overall, our findings highlight a previously unrecognised role of AATF in GBM proliferation and mitochondrial metabolism supporting its potential as a target for therapeutic intervention.

## Introduction

Cancer cells adapt their metabolism to meet the energetic requirements necessary to support their constant growth and division. Extensive research has clearly demonstrated that a metabolic switch characterised by increased glycolysis and decreased oxidative phosphorylation (OXPHOS), a phenomenon known as Warburg effect or anaerobic glycolysis, is a feature of tumorigenesis [1, 2]. However, an increasing body of evidence is now showing that many tumours, including glioblastoma multiforme (GBM), are heavily reliant on the OXPHOS pathway for their proliferation, survival and chemotherapy resistance [3–6]. Consistently, upregulation of mitochondrial genes and proteins responsible for OXPHOS has been demonstrated in many malignant tumours [7–9]. GBM, the higher-grade malignant glioma (grade IV), represents the most common and aggressive kind of primary brain tumour in adults [10–12], with a median survival of less than 15 months [13]. The current management of GBM includes safe surgical resection and post-operative radiotherapy and chemotherapy [14–16]. Unfortunately, this therapeutic approach fails to give long-term remission for patients, due in part to the high molecular and metabolic heterogeneity of GBM cells. About this, recent reports have shown the existence of multiple metabolic dependencies in GBM, and mitochondrial respiration has been shown to play a crucial role in GBM tumorigenesis [17, 18]. In line with these findings, Garofano et al., through single-cell RNA expression profiling, identified a mitochondrial subtype of GBM tumours that exhibits increased reliance on the OXPHOS pathway and enhanced sensitivity to inhibitors of mitochondrial metabolism [19]. For all these reasons, targeting mitochondrial respiration is emerging as an attractive anti-cancer strategy. Indeed, suppression of the OXPHOS pathway by pharmacological inhibition of the electron transport chain (ETC) has already been shown to inhibit GBM cells proliferation [20–22]. Nuclear Respiratory Factor 1 (NRF-1) is the master regulator of mitochondrial biogenesis and respiratory functions through its ability to regulate the expression of the OXPHOS genes as well as nuclear-encoded genes involved in mitochondrial DNA transcription, such as mitochondrial transcription factor A (TFAM) [23, 24]. Furthermore, emerging evidence supports its involvement in key cellular processes, including cell cycle regulation, apoptosis, response to stress and in the progression of different types of cancer [25, 26]. Interestingly, NRF-1 expression has been associated with the severity of astrocytoma and poor prognosis in GBM [27, 28]. Che-1/Apoptosis Antagonising Transcription Factor (AATF) is an RNA polymerase (RNA Pol) I and II binding protein [29, 30], mainly involved in transcriptional regulation [31–33]. AATF interacts with several transcription factors and regulatory proteins influencing their activities and modulating the expression of genes involved in cell cycle progression, apoptosis and response to stress [32, 34–37]. It predominantly shows nuclear and nucleolar localisation [30, 38, 39], but its presence has also been detected in centrosomes, Golgi apparatus, cytoplasm and mitochondria [40–42]. Furthermore, AATF has been shown to prevent apoptotic cell death by safeguarding mitochondrial function and reducing oxidative damage in human kidney proximal tubule cells [41]. To date, this protein has been involved in the pathogenesis of several types of cancer, including multiple myeloma, B-cell precursor acute lymphoblastic leukaemia (BCP-ALL), hepatocellular and lung carcinomas [43–46], but its role in the biology of GBM and the pathways in which it takes part are not completely clarified. In this study, we show that AATF depletion is associated with cell cycle arrest in several GMB cell lines, reduced mitochondrial respiration and altered mitochondrial architecture. Moreover, AATF-depleted cells show a significant reduction in their ability to form colonies, in vitro and to develop tumours, in vivo. Mechanistically, we provide evidence that AATF interacts with NRF-1 and that it is required to promote NRF1-dependent transcription of the OXPHOS genes by modulating chromatin condensation and RNA polymerase II recruitment. Overall, we identify AATF as a new regulator of GBM cell proliferation through its ability to regulate the OXPHOS programme and to maintain mitochondrial homoeostasis.

## Materials and methods

### Cell lines, transfections and reagents

Human U87, U138, SW1783, LN229 and U251 cell lines were cultured in Dulbecco’s Modified Eagle Medium (DMEM, Euroclone) supplemented with 10% inactivated foetal bovine serum (FBS, Thermo Fisher Scientific), 2 mM glutamine (Thermo Fisher Scientific) and 40 μg/ml gentamicin. All cell lines were cultured at 37 °C, in a humidified atmosphere with 5% CO_2_. Mycoplasma contamination was periodically checked by polymerase chain reaction (PCR) analysis, using the following primers:

Forward: 5’ - ACTCCTACGGGAGGCAGCAGTA - 3’

Reverse: 5’ - TCGACCATCTGTCACTCTGTTAAC - 3’

Transfection experiments were carried out using Lipofectamine 3000 Transfection System (Thermo Fisher Scientific) according to the manufacturer’s instructions. Cells were analysed 72 hours after transfection by western blot (WB) or quantitative real-time PCR. Stealth siRNA oligonucleotides targeting AATF (siAATF #1, cat. n. 1299003-HSS120157 and siAATF #2, cat. n. 1299003-HSS120158) and NRF-1 (siNRF-1 cat. n. 1299003-HSS107320, HSS107321, HSS107322) or a control sequence (siControl, cat. n. 12935300) were purchased from Thermo Fisher Scientific. AATF-myc expressing vector has already been described [30].

### Proximity ligation assay (PLA)

U138 cells were seeded in a 96-well PhenoPlate (Perkin Elmer, USA), fixed with 4% formaldehyde for 10 min at room temperature, permeabilized with 0.1% Triton X-100 in phosphate-buffered saline (PBS) for 5 min at room temperature, and processed according to the Duolink In Situ proximity ligation assay (PLA) protocol (Sigma-Aldrich). Anti-NRF-1 (Diagenode, C15200013; 1:200) and anti-AATF (Bethyl A301-032A; 1:200) were used as primary antibodies. Negative controls were performed by omitting NRF-1 primary antibody or transfecting cells with specific siRNA oligonucleotides targeting AATF or NRF-1. Nuclei were visualised by staining with 1 μg/ml Hoechst dye 33258 (Sigma-Aldrich). Cells were subjected to confocal imaging analysis using Opera Phenix high-content-screening system (Perkin Elmer, USA) equipped with Harmony ^TM^ high content imaging and analysis software (Perkin Elmer, USA, version 4.8).

### Cellular extracts and immunoprecipitation

Total cellular extracts were prepared as previously described [32]. For immunoprecipitation experiments, nuclear extracts were pre-cleared by incubation with protein A/G conjugated to agarose beads (Thermo Fisher Scientific) for 1 h at 4 °C on a rotating wheel. Pre-cleared nuclear cellular extracts were collected by centrifugation and incubated overnight at 4 °C on a rotating wheel, with the indicated antibodies. Protein A/G conjugated to agarose beads was then added to the samples and the incubation was continued for an additional hour. Immuno-complexes were collected by centrifugation, washed five times with dilution buffer (50 mM TRIS pH 7.4, 150 mM NaCl, 5 mM EDTA, 10 mM NaF, 0.5% NP-40) and eluted in lithium dodecyl sulphate (LDS) sample buffer (Thermo Fisher Scientific) for WB analyses. All buffers were supplemented with protease and phosphatase inhibitors (1 μg/ml aprotinin, 1 μg/ml leupeptin, 1 mM Na_3_VO_4_, 10 mM PMSF).

### Western blotting

Western blotting (WB) analysis of protein samples was carried out as previously described [32]. Briefly, samples were separated by electrophoresis and transferred onto nitrocellulose membranes. Membranes were blocked in 5% Nonfat-Dried Milk (NFDM) in 0.1% Tween-PBS and incubated overnight at 4 °C with the appropriate primary antibodies. After three washes with 0.1% Tween-PBS, membranes were incubated with the appropriate HRP-linked secondary antibodies (Bio-Rad) at room temperature for 45 min. Following three additional washes with 0.1% Tween-PBS, membranes were analysed by chemi-luminescence (GE Healthcare Life Science). Images were acquired using Alliance Mini HD6 system by UVITEC Ltd, Cambridge, equipped with UVI1D Software (UVITEC HD, 14-630275, Cambridge, UK). Antibodies used in this study are listed in Table S1.

### Clonogenic survival assay

U138 cells depleted or not for AATF expression were seeded at a density of 1000 cells/well into 6-well dishes. Fresh medium was added every 4 days. After 7–14 days, colonies were stained with 0.5% crystal violet (Sigma Aldrich) and manually counted.

### Senescence assay

Cell senescence was evaluated by Senescence Associated (SA) β-galactosidase hydrolysis using CellEvent Senescence Green Detection Kit (Thermo Fisher Scientific) according to the manufacturer’s instructions.

### In vivo experiments

All procedures involving mice and their care were authorised and certified by the decree n. 171/2025-PR and conformed to the relevant regulatory standards in accordance with the Italian legislation. For transplantation experiments, U138 cells were transfected with either control siRNA (U138 siControl) or AATF siRNA (U138 siAATF) using Lipofectamine 3000, according to the manufacturer’s instructions. After 24 h, 3 × 10^6^ U138 siControl or U138 siAATF cells (in 50% Matrigel, Corning, NY, USA) were subcutaneously injected in the right flank of 8-week-old male NOD/SCID (NOD.CB17-Prkdc scid/NCrHsd) mice (Envigo RMS s.r.l.) (*n* = 4 per group). Animals were observed daily, weighted two times/week and tumour volume (mm^3^) calculated as length × width^2^/2. At the end of the treatment (5 weeks), the tumours were collected and analysed by western blot and Immunohistochemical analysis. All the experiments and analyses were performed in a blinded fashion.

### Immunohistochemical analysis

Tumours’ mice were processed by the CellientTM Automated Cell Block System (Hologic Corporation, Marlborough, MA), which is fully automated and creates a paraffin-embedded cell block using isopropanol for dehydration and xylene for clarification. FFPA samples were cut at 3 μm using a microtome LEICA SM 2000R (Advanced Research Systems Inc., Macungie, PA) and mounted onto slides. Subsequently, the slides were dewaxed in xylene and rehydrated through a series of graded ethanol solutions and stained with Gill’s Hematoxylin (Bio-optica, Milan) and Eosin (Bio-optica, Milan). The slides were incubated with primary antibody for Ki-67 (clone MM1-L-CE LEICA) in an automated immuno-stainer (Bond-III, LEICA, Biosystems, Italy), according to the manufacturer’s instructions and the images were then captured at ×20 magnification using Aperio Image Scope system equipped with a Digital Image Capture software. The evaluation was based on the percentage of positive cells.

### Flow cytometry

Cells depleted or not for AATF expression and cells cultured or not for 24 or 72 h in serum-free media, were trypsinized and re-suspended in ice-cold PBS at a density of 1 × 10^6^ cells/ml, fixed by adding 2 ml of ice-cold 70% ethanol in PBS while vortexing, and incubated at least 30 min on ice. Next, cells were collected by centrifugation at 1000 × *g* and treated with RNase A 1 mg/ml (Thermo Fisher Scientific) at 37 °C for 30 min. Finally, cells were stained with propidium iodide (Sigma-Aldrich) and incubated in darkness for 60 min or overnight before analysis. Samples were analysed by flow cytometry using Attune Flow Cytometer (Thermo Fisher Scientific). A total of 10,000 cells were counted for each sample, and gating and quantification of cell population were carried out using FlowJo software.

### MTT assay

Cell viability was measured using the 3-(4, 5-dimethylthiazol-2-yl)-2, 5-diphenyl tetrazolium bromide (MTT) (Sigma-Aldrich, cat. n. M5655) assay according to the manufacturer’s instructions. Briefly, cells depleted or not for AATF expression were plated in 96-well plates at a concentration of 2 × 10^5^ cells/well, in triplicate. After 24 h of culture, 0.5 mg/ml of MTT was added to each well. After 3 h of incubation at 37 °C, coloured crystals of formazan were dissolved with 100 µl of DMSO (Sigma-Aldrich), and absorbance was read at 570 nm using Multiskan FC Microplate Photometer (Thermo Fisher Scientific, USA).

### Mito stress test and ATP rate assay

U87 and U138 cells depleted or not for AATF expression were seeded in Seahorse XF cell culture plates at a density of 1 × 10^5^ cells/well the day before the assay. The day of the assay, after a 30-min calibration of the XF sensor with the pre-incubated sensor cartridge, the cell plate was loaded into the Seahorse XF HS Mini Analyser (Agilent Technologies). Oxygen Consumption Rate (OCR) and ATP rate were measured in real-time with Mito Stress Test kit (Agilent, AG-103010-100) and ATP Rate Assay Kit (Agilent), respectively, under basal conditions and after sequential injection of the complex inhibitors oligomycin (1.5 µM), FCCP (1.2 µM), and a mixture of rotenone and antimycin A (0.5 μM each). Subsequently, cells were stained with Hoechst dye 33258 (Sigma-Aldrich) and cell count of each well was determined by imaging the cells using Cell Profiler. Data are presented as mean ± standard deviation (SD) of three independent experiments.

### Mitochondrial morphology analysis

U138 cells depleted or not for AATF expression were seeded in a 96-well PhenoPlate (Perkin Elmer, USA) and cultured overnight. After this time, mitochondrial morphology was examined following staining with Mito Tracker Red CMXROS (Thermo Fisher Scientific, M7512) in presence of 20 μg/ml Hoechst dye 33258 (Sigma-Aldrich) for 30 min at 37 °C. Cells were then washed twice with PBS and analysed using Opera Phenix High-content System equipped with Harmony^TM^ high content imaging and analysis software (Perkin Elmer, USA, version 4.8). Confocal images acquisition was performed using a 63× objective. To analyse the different organisation of mitochondrial network, we measured the Saddle, Edges, and Ridges (SER) features of the 568-channel image of mitochondria, a set of texture properties. This allowed us to distinguish between mitochondrial network with a filamentous texture, and mitochondrial network with a ring-like morphology. Briefly, using the PhenoLOGIC Harmony software, individual cells were identified using the “Find Nuclei” building block, where nuclei were stained with Hoechst dye. Meanwhile, the “Find Cytoplasm” building block was used to delineate the mitochondrial network, with mitochondria stained using MitoTracker dye. Finally, we calculated the positional properties of this network by excluding the nuclear area and determined the fluorescence texture properties using the SER features method.

### Mitochondrial superoxide detection

U138 cells depleted or not for AATF expression were seeded at a density of 2 × 10^3^ cells/well in a 96-well PhenoPlate (Perkin Elmer, USA) and allowed to adhere for 24 h under normal cell culture conditions. Next, to assess mitochondrial ROS production, cells were washed twice with PBS and subsequently incubated with 5 µM MitoSOX Red dye (Thermo Fisher Scientific, USA) for 25 min at 37 °C. Cells were washed twice with PBS again and the fluorescence intensity was measured at a 488 nm excitation wavelength and 525 nm emission wavelength using Opera Phenix High-content System (Perkin Elmer, USA) and analysed with Harmony^TM^ high content imaging and analysis software (Perkin Elmer, USA, version 4.8). For the experiments with the mitochondria-targeted antioxidant MitoTEMPO, U138 cells depleted or not for AATF expression were treated or not with 100 µM of the reagent for 24 h before analysis.

### RNA isolation and quantitative real-time PCR

Total RNA was isolated from cells depleted or not for AATF expression using NucleoZOL reagent (Macherey-Nagel) according to the manufacturer’s instructions. cDNA was synthesised from an equal amount of RNA by reverse transcription using M-MLV reverse transcriptase (Thermo Fisher Scientific) and then used to perform quantitative real-time PCR (q-RT-PCR) with specific primers using a PowerUP SYBR Green Master Mix (Applied Biosystems) on a 7500 Fast Real-Time PCR System (Applied Biosystems), following the manufacturer’s instructions. A melting curve analysis was performed to confirm that single products were amplified, and data were processed using the 7500 software v2.0.6 (Applied Biosystems). Relative fold changes were determined by the comparative threshold (ΔΔCT) method [47] using β-actin as endogenous normalisation control. Data are presented as mean ± SD of three independent experiments, performed in duplicate. Specific primer pairs employed in q-RT-PCR amplifications are listed in Table S2.

### Chromatin immunoprecipitation assay

Chromatin immunoprecipitation (ChIP) experiments were performed as previously described by Bruno et al. [48] using the following antibodies: anti-AATF (Bethyl A301-032A), anti-H3K27Ac (Millipore, 07-360), anti-RNA polymerase II (phospho S5) (Abcam, ab5131), anti-H3K9me3 (Abcam, ab8898). Immunoprecipitations with no specific immunoglobulins (Santa Cruz Biotechnology) were performed as negative controls. For quantitative ChIP analysis (ChIP-qRT), 1 µl of purified DNA was used for amplification on a 7500 Fast Real-time PCR System (Applied Biosystems) using a PowerUP SYBR Green Master Mix (Applied Biosystems). Primer pairs used in these experiments are listed in Table S2.

### RNA-seq

Total RNA was extracted using Qiazol (Qiagen, IT), purified from DNA contamination through a DNase I (Qiagen) digestion step and further enriched by Qiagen RNeasy columns specific for gene expression profiling (Qiagen). Quantity and integrity of the extracted RNA were assessed using a NanoDrop Spectrophotometer (NanoDrop Technologies) and a TapeStation System (Agilent), respectively. RNA libraries for sequencing were generated in triplicate using the same amount of RNA for each sample according to the Illumina Stranded Total RNA Prep kit, with a first ribosomal depletion step using Ribo-Zero Plus (Illumina). The libraries were quantified by q-RT-PCR and sequenced in paired-end mode (2 × 100 bp) with NovaSeq 6000 System (Illumina). For each sample generated by the Illumina platform, a pre-process step for quality control was performed to assess sequence data quality and to discard low-quality reads.

### ChIP-sequencing

ChIP-sequencing (ChIP-seq) experiment was performed following the ChIPmentation protocol [49] in duplicate using a specific NRF-1 Antibody (Diagenode, C15200013). The final libraries were controlled on TapeStation system (Agilent) and sequenced on a NovaSeq 6000 System (Illumina) using 2 × 100 cycles in paired-end mode.

### Computational methods

#### RNAseq analysis

Samples were aligned to the reference genome hg19 using STAR, and transcript quantification was performed with RSEM, employing the nf-core/rnaseq pipeline (v. 3.12.0) with Nextflow (v. 24.10.4.5934) with default parameters. The resulting quantification files were normalised using the standard edgeR (v. 4.0.16) pipeline. Normalised counts were then used to perform differential expression analysis, selecting genes with *q*-value < 0.05 and |log₂FC| > 0.7. Gene ontology analysis was conducted for genes with –1.4 ≤ log₂FC ≤ –0.7 using the ClusterProfiler R package (version 4.10.1), with the enrichKEGG function (parameters: organism = ‘hsa’, pvalueCutoff = 0.1, qvalueCutoff = 0.05). Conversion from gene name to gene ID was performed with the bitr function from ClusterProfiler. The same procedure was also performed for up-regulated genes (0.7 ≤ log_2_FC ≤ 1.4). Conversion from gene name to ID was carried out using the bitr function from ClusterProfiler.

#### ChIP-seq analysis

Samples were aligned to the reference genome hg19 with Bowtie2 and peaks were called with MACS2 available in the nf-core/atac-seq pipeline (v2.0) with Nextflow (v. 24.10.4.5934), using default parameters. Significant peaks were identified and intersected using BEDTools intersect (v. 2.27.1) with standard parameters to obtain lists of common and condition-specific peaks for NRF-1 under two conditions: siControl and siAATF. To quantify signal intensities in each peak set, deepTools (v. 3.5.3) was used with the following functions: computeMatrix reference-point with default parameters and plotHeatmap with default parameters. Venn diagrams were produced intersecting control and interfered bed files with the venn function from the Intervenn tool (v. 0.6.5). Peak visualisations were generated from bigWig files using Integrative Genomics Viewer (IGV) v. 2.14.1.

### Statistical analysis

All the in vitro experiments mentioned here were performed three times (biological replicates). For the in vivo studies, 4 mice were used in each experimental group. The data presented here were as mean ± standard deviation (SD). Statistical analyses were performed using two-tailed Student’s *t* tests or two-way ANOVA test. In experiments in which the control was set to 1, statistical analyses were performed using the Mann–Whitney test. Statistical significance is indicated by asterisks as follows: *P < 0.05, **P < 0.01, ***P < 0.001, n.s. not significant.

## Results

### AATF promotes proliferation of GBM cells, in vitro and in vivo

**Fig. 1:**
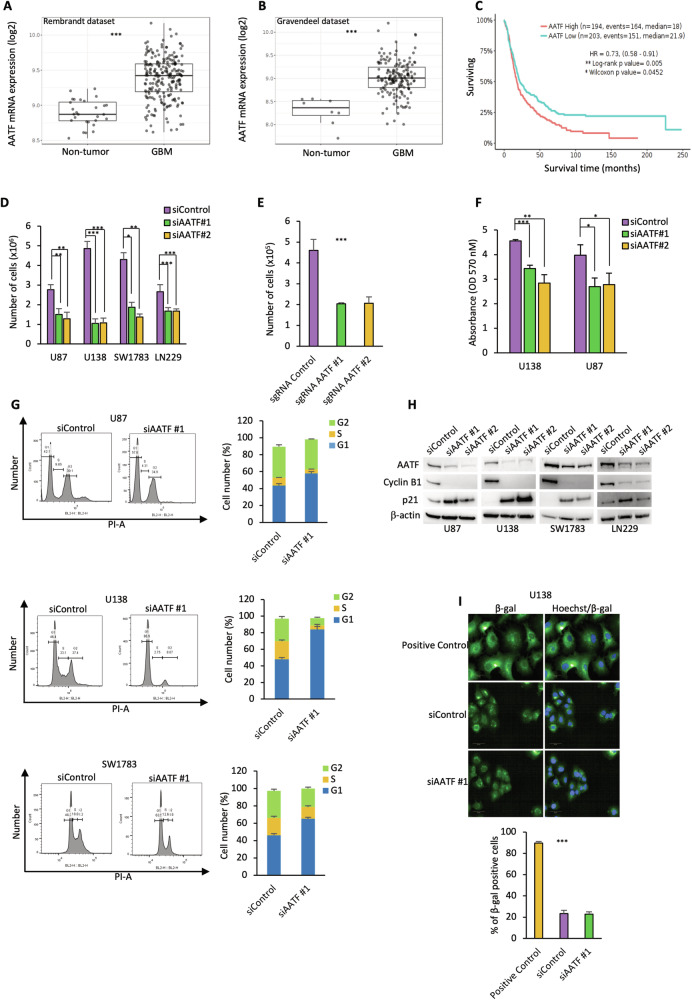
AATF promotes proliferation of GBM cells in vitro. Analysis of AATF expression in Rembrandt (**A**) (GBM *n* = 219, non-tumour *n* = 28) and in Gravendeel (**B**) (GBM *n* = 159, non-tumour *n* = 8) patient datasets. **C** Kaplan-Meier curve generated on data from GlioVis data portal (Rembrandt dataset), showing significant correlation between AATF expression and poor patient prognosis in GBM patients compared to controls. Proliferation assays of the indicated cell lines transfected with two different siRNA oligonucleotides targeting AATF (siAATF #1-siAATF #2) or a control sequence (siControl) for 72 h (**D**) or of U138 cells stably expressing the dCas9-KRAB transcriptional repressor complex transiently transfected with Control or AATF targeting sgRNAs (**E**), were performed by counting the cells with Countess 3 Cell Counter (Thermo Fisher Scientific). **F** Cell viability was evaluated using MTT assay in U138 and U87 cells treated as in (**D**). **G** Flow cytometry analysis of cell cycle distribution of U87, U138 and SW1783 cells treated as in D. DNA content was measured by propidium-iodide staining (left panels). Percentages of cells in each phase of the cell cycle were measured by FlowJo software (right panels). **H** WB analysis with the indicated antibodies of total cell extracts from cell lines treated as in D. β-actin was used as loading control. **I** Top: representative fluorescence images of U138 cells subjected to the β-galactosidase (β-gal) assay. Green fluorescence indicates β-galactosidase–positive cells, while nuclei are counterstained with Hoechst dye. Images were acquired with confocal Opera Phenix High Content Screening System using a 40× air objective. Bottom: quantification of β-gal-positive cells was calculated using Harmony analysis software. Positive control was performed by treating cells with Adriamycin (100 nM for 7 days). Data are presented as the mean ± standard deviation (SD) of three independent experiments. *p ≤ 0.05, **p ≤ 0.01, ***p ≤ 0.001, n.s. = not significant. See also Supplementary Figs. S1 and S2.

**Fig. 2:**
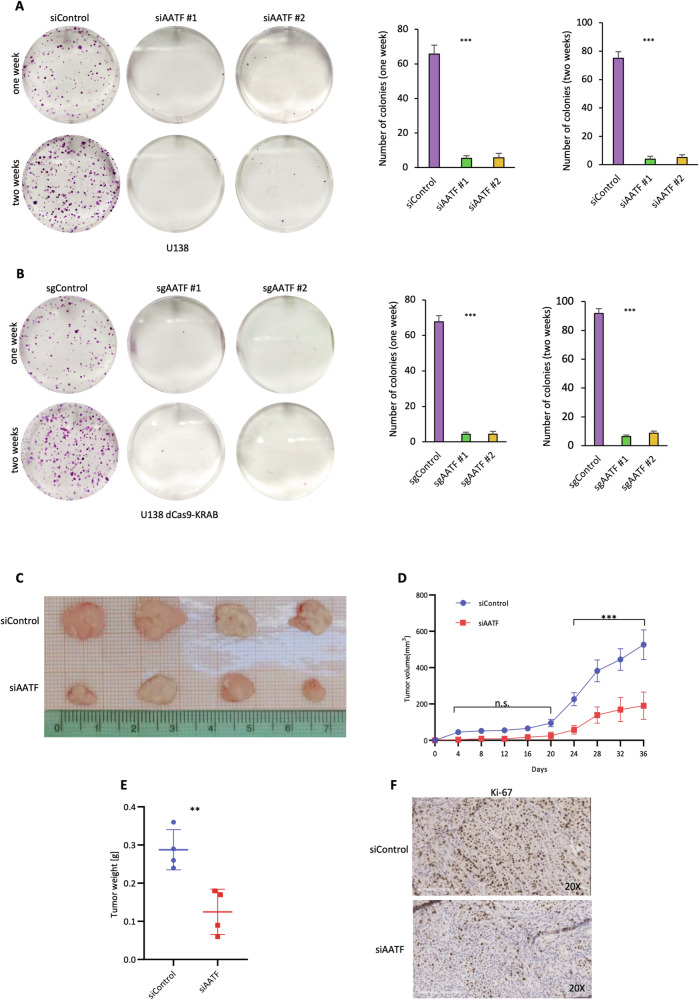
AATF promotes proliferation of GBM cells, in vivo. Representative plates of clonogenic assays (left) and relative quantification of colony numbers (right) performed on U138 cells after transfection with control siRNA (siControl) or two distinct siRNAs targeting AATF (siAATF #1 and siAATF #2) (**A**) or on U138 cells stably expressing the dCas9-KRAB transcriptional repressor complex and transiently transfected with Control or AATF targeting sgRNAs (**B**). 72 h after transfection, cells were counted and re-plated at low density to allow colony formation for one or two weeks. Colonies were then fixed and stained with crystal violet. **C** Tumours were established by injecting cells into the right flank of nude mice, and images were taken on day 36 post-implantation. At the study endpoint, tumours were surgically removed and photographed on a metric grid for size comparison. The image shows subcutaneous tumours from each experimental group (*n* = 4), illustrating the overall difference in tumour size between siControl and siAATF mice. The centimetre ruler provides a visual reference for tumour dimensions. **D** Tumour volume was calculated from caliper measurements every three days starting from day 4 after the inoculation of the transfected cells. The line graph shows mean tumour volume ± SD for each experimental group (blue circles: siControl; red squares: siAATF). Statistical analysis was performed using two-way ANOVA with repeated measures. **E** Graph showing tumour weights at the experimental endpoint. Points represent individual tumours, and horizontal bars indicate mean ± SD. AATF-silenced tumours exhibited a significantly lower weight compared with the control group, as determined by an unpaired two-tailed t-test. **F** Representative IHC images of explanted tumours stained with primary antibody for Ki-67 (clone MM1-L-CE LEICA) and then captured at ×20 magnification using Aperio Image Scope system equipped with a Digital Image Capture software. Data are presented as the mean ± SD of three independent experiments. *p ≤ 0.05, **p ≤ 0.01, ***p ≤ 0.001, n.s. not significant. See also Supplementary Fig. S3.

It has previously been shown that AATF is involved in the development and progression of both solid and haematological malignancies [43–46]. However, its role in GBM has not yet been fully elucidated. In line with a recent report showing a notable upregulation of AATF in almost all the analysed cancer types, including GBM [50], the analysis of the Rembrandt (GBM *n* = 219, non-tumour *n* = 28) and the Gravendeel datasets (GBM *n* = 159, non-tumour *n* = 8) available on the GlioVis data portal, confirmed that AATF expression is upregulated in samples from GBM patients compared to non-tumour brain tissue samples (Fig. 1A, B). In addition, its expression correlates with tumour grade and higher AATF levels are observed in the pro-neural, classical and mesenchymal subtypes compared with the neural one, suggesting its possible involvement in tumour aggressiveness [51] (Fig. S1A). Consistently, a Kaplan-Meier survival analysis on the Rembrandt dataset revealed that AATF overexpression is associated with poorer prognosis (Fig. 1C), suggesting an important role for this protein in the biology of GBM. To elucidate the functions of AATF in this malignancy, we first analysed the effect of its depletion on proliferation and survival of glioblastoma cells. To this aim, we transfected several GBM cell lines with two siRNA oligonucleotides targeting different regions of AATF mRNA (siAATF#1 and siAATF#2) or with a control sequence (siControl). As shown in Fig. 1D, downregulation of AATF expression is associated with a strong decrease in cellular proliferation, in all the analysed cell lines when compared with control condition, with both siRNA oligonucleotides. Similar results were obtained by silencing its expression using the CRISPRi genome editing system [52] (Figs. 1E and S1B). The ability of AATF to promote GBM cellular proliferation was further confirmed by MTT assay, which estimates cell proliferation based on metabolic activity (Fig. 1F). In addition, flow cytometric analysis (FACS) revealed that GBM cells depleted for AATF expression show a significant decrease in the percentage of cells in S and G2/M phases of the cell cycle, with a concomitant increase in G1 phase cells, similarly to the pattern observed upon serum deprivation-induced cell cycle arrest, suggesting an involvement of AATF in the regulation of G1/S transition (Figs. 1G and S1C, D). Consistent with these results, AATF inhibition induces an increase in p21 expression, a G1 phase regulator, with a parallel decrease in cyclin B1 levels, a specific G2 phase marker (Figs. 1H and S1E). Consistent with these results, a decrease in the phosphorylation level of Akt and Erk was also observed (Fig. S1F). Notably, AATF appeared to be able to control cell proliferation without affecting cell viability or senescence induction, as shown by the percentage of viable cells assessed through trypan blue staining (Fig. S2A, B), the absence of the typical marker of apoptosis cleaved Caspase-7 (Fig. S2C), as well as Annexin V assay and β-gal staining (Figs. S2D and 1I). To further investigate the role of AATF in GMB cell proliferation, we performed long-term clonogenic survival assays. AATF depletion resulted in a marked reduction in the number and size of colonies compared to control cells (Figs. 2A, B and S3A, B), and enhanced the growth-inhibitory effect of the chemotherapy agent temozolomide (Fig. S3C, D), thus confirming its important role in sustaining cell proliferation. The reduced proliferative capacity observed upon AATF silencing in vitro was further supported by in vivo experiments. Tumours arising from AATF-depleted U138 cells (Fig. S3E) injected subcutaneously in NOD/SCID mice exhibited significantly diminished growth and proliferative activity compared to control tumours (Fig. 2C–E). These findings demonstrate that the loss of AATF impairs the tumour-forming ability of GBM cells within the in vivo microenvironment, reinforcing the notion that AATF is essential for sustaining GBM progression [53]. Consistent with these findings, Ki-67 staining of explanted tumour tissues revealed a lower proportion of Ki-67–positive nuclei in those arising from AATF-depleted cells, indicating a reduced proliferative activity compared with control tumours (Figs. 2F and S3F). Together, these results strongly suggest a role for AATF in the regulation of GBM cell proliferation and progression both in vitro and in vivo.

### AATF regulates OXPHOS gene expression in GBM cells

**Fig. 3:**
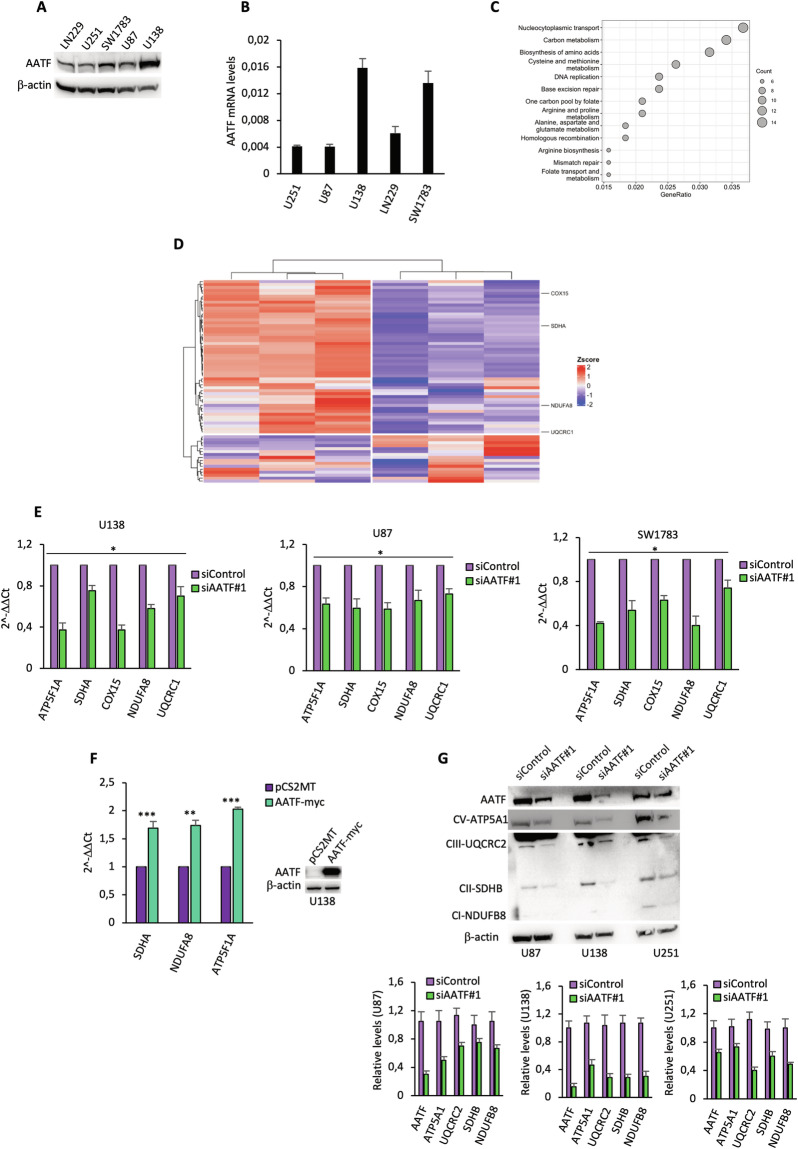
AATF regulates OXPHOS gene expression in GBM cells. **A** Representative WB of AATF expression in total cell extracts of different GBM cell lines. Proteins extracted from the same number of cells were loaded. β-actin was used as loading control. **B** qRT-PCR analysis of AATF mRNA levels in the indicated cell lines. The quantification of the absolute expression of AATF mRNA was obtained by the comparative Ct method (2^−ΔCt^ method) [68]. **C** The scatterplot represents the ontologies enrichment analysis of siAATF down-regulated genes. The *x*-axis represents the GeneRatio (the proportion of input genes associated with each GO term), dot size reflects the number of genes contributing to the term. **D** Heatmap derived from the intersection of RNAseq-detected downregulated genes and an OXPHOS-associated signature. Unsupervised clustering divides samples into two clades according to the Z-score (TMM) value of gene expression. **E** qRT-PCR analysis performed in the indicated GBM cell lines transfected with siRNA targeting AATF (siAATF #1) or with a siRNA for a control sequence (siControl), to validate the downregulation of the selected OXPHOS genes showed in the heatmap. **F** qRT-PCR (right panel) and relative WB analyses performed on U138 cells transfected with AATF-myc expressing vector or with a control vector (pCS_2_MT). **G** Top: representative WB with the indicated antibodies of total cellular extracts from GBM cell lines treated as in E. Bottom: bar plot showing the expression of the indicated proteins calculated by densitometric analysis of three independent experiments. *p ≤ 0.05, **p ≤ 0.01, ***p ≤ 0.001, n.s. not significant.

AATF is involved in many cellular processes through its ability to act as a transcriptional co-factor [37, 54]. To shed light on the cellular pathways underlying AATF’s ability to sustain GBM cell proliferation, we performed an RNA-seq analysis to profile the transcriptome signature in response to AATF silencing. This analysis was performed in the U138 cell line, which expresses the highest AATF level among all the cell lines analysed (Fig. 3A, B), and downregulation of AATF was confirmed by WB analysis (Fig. S4A). Differential expression analysis resulted in a total of 2427 modulated genes, including 1258 up-regulated (*q*-value < 0.05 and log2FC > 0.7) and 1169 down-regulated transcripts (*q*-value < 0.05 and log2FC < −0.7) (Fig. S4B). Gene Ontology analysis of the most up-regulated genes revealed an enrichment of pathways, i.e. PI3K–Akt, calcium signalling, lysosomal and AGE–RAGE pathways, which collectively suggest the activation of stress-response and pro-survival programmes capable of sustaining cell viability even under the cell-cycle arrest induced by AATF silencing (Fig. S4C). On the other hand, pathways essential to sustain the proliferation of rapidly dividing cells, including carbon metabolism and amino acids biosynthesis, were found down-regulated in response to AATF depletion (Fig. 3C). In particular, the intersection of the RNA-seq-downregulated genes and an OXPHOS-associated signature, revealed that AATF depletion is associated with a consistent downregulation of the expression of most genes of this signature (53 out of 66) (Fig. 3D). Interestingly, when we compare our signature with RNAseq experiments conducted in KMS27 [46] and BIU87 (GSE248071) cell lines depleted for AATF expression, we did not observe any overlap, suggesting that the modulation of OXPHOS signature occurs specifically in glioblastoma cells (Fig. S4D). The downregulation of five of the OXPHOS genes, indicated in Fig. S4E, was confirmed by q-RT PCR in different GBM cell lines depleted or not for AATF expression (Figs. 3E and S4F). Conversely, over-expression of AATF resulted in an increased expression of these genes (Fig. 3F). Moreover, immunoblot analysis with a Total OXPHOS Antibody Cocktail kit, containing antibodies against one subunit from each of the five complexes (I—V) of the OXPHOS system, revealed that AATF silencing leads to a decrease in the amount of all the components of the OXPHOS complexes compared to control (Fig. 3G), supporting the functional relevance of this protein in regulating mitochondrial respiration. In agreement with the role of AATF in sustaining OXPHOS gene expression, in silico analysis highlighted a positive correlation between AATF and OXPHOS gene expression in GBM patients (Fig. S4G).

### AATF sustains mitochondrial respiration in GBM cells

**Fig. 4:**
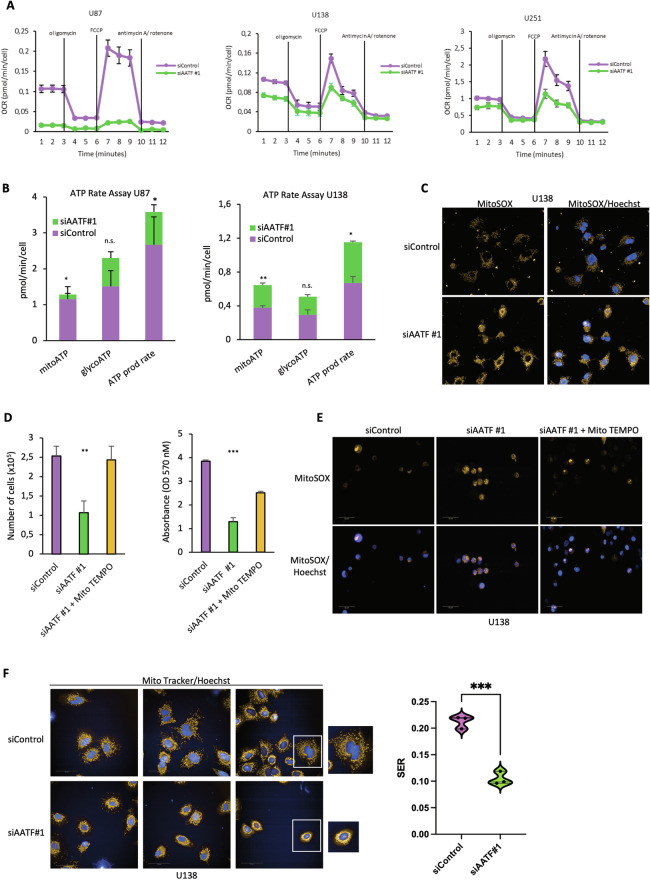
AATF sustains mitochondrial respiration in GBM cells. **A** Different GBM cell lines transfected with AATF siRNA #1 or negative control were analysed using Cell Mito Stress Test, 72 h after transfection. OCR was measured followed by the consecutive injections of oligomycin (1 µM), FCCP (0.5 µM), and antimycin A (0.5 µM)/rotenone (0.5 µM), and normalised to manually counted cells. **B** ATP production rates calculated for U87 and U138 cells treated as in A (*n* = 3 biological replicates). **C** Representative confocal images of U138 cells treated as in A and stained with the mitochondrial superoxide indicator MitoSOX and acquired with confocal Opera Phenix High Content Screening System using a 40x air objective. Nuclei were counterstained with Hoechst dye. **D** Left: proliferation assay performed by counting, with Countess 3 Cell Counter (Thermo Fisher Scientific), U138 cells depleted or not for AATF expression and treated or not with Mito TEMPO 100 µM for 24 h before analysis. Right: cell viability was evaluated using MTT assay in the same cells described above. **E** Representative confocal images of U138 cells treated as in (**D**) and stained with Mito SOX and Hoechst dye and acquired with confocal Opera Phenix High Content Screening System using a 40× air objective. **F** Left: representative confocal images of U138 cells treated as in A and stained with Mito Tracker Red CMX Ros (mitochondria labelling) and Hoechst dye (nuclei staining). Automated confocal microscopy was performed using Opera Phenix High Content Screening System with a 63× objective. Right: quantification of mitochondria morphological changes using SER texture analysis on Harmony analysis software and represented using violin plots. Data are presented as the mean ± SD of three independent experiments. *p ≤ 0.05, **p ≤ 0.01, ***p ≤ 0.001, n.s. not significant.

The downregulation of OXPHOS gene expression observed upon AATF depletion prompted us to assess its activity on mitochondrial functions and structure, starting with the analysis of mitochondrial respiration. To this aim, oxygen consumption rate (OCR), a key indicator of mitochondrial respiration, was evaluated in U87, U138 and U251 cells depleted or not for AATF expression (Fig. S5A), using the Seahorse Mito Stress Test. Interestingly, AATF-depleted cells displayed a significantly lower respiration both at basal level and upon treatment with selective mitochondrial inhibitors (Figs. 4A and S5B). In agreement with these results, ATP levels were also decreased in AATF-silenced GMB cells compared to control ones (Fig. 4B). Significantly, in accordance with previous reports [41, 43, 55–57], we found that AATF silencing induces a significant increase in the amount of reactive oxygen species (mROS), detected by staining with the specific mitochondrial-ROS probe MitoSox and with the fluorescent probe DCFDA (Figs. 4C and S5C, D). Moreover, treatment of AATF-depleted cells with the ROS scavenger MitoTempo, partially rescued the proliferation arrest induced by AATF depletion (Figs. 4D and S5E) while simultaneously reducing ROS accumulation (Figs. 4E and S5F), supporting a role for mitochondrial ROS in mediating the effects of AATF loss. Since OXPHOS gene expression is closely linked to mitochondrial activity, influencing both structural and functional organisation [58], we analysed mitochondrial architecture in U138 cells depleted or not for AATF expression. To this aim, we stained GBM cells with Mitotracker Red CMXRos, a specific dye that passively diffuse across the plasma membrane and accumulate in active mitochondria. Notably, while most of the control cells display a filamentous, interconnected mitochondrial network, upon AATF-depletion we observed, similarly to the effect of the respiratory poison, antimycin, an increase of cells exhibiting a “ring-shaped” mitochondrial network, considered a typical hallmark of mitochondrial dysfunction [59–61], and a concomitant decrease of Saddle, Edges, and Ridges (SER) values, indicative of a change in mitochondrial morphology (Figs. 4F and S6A, B). Furthermore, expression analysis of genes involved in the regulation of mitochondrial fusion and fission events revealed that AATF depletion is associated with up-regulation of the fission-related genes FIS1, DNM1L and MFF, in line with the notion that enhanced ROS accumulation triggers mitochondrial fragmentation (Fig. S6C). Altogether, these results clearly show that AATF is required for efficient mitochondrial respiration and maintenance of a proper mitochondrial network in GBM cells.

### AATF binds to the OXPHOS gene promoters and promotes their transcription through NRF-1

**Fig. 5:**
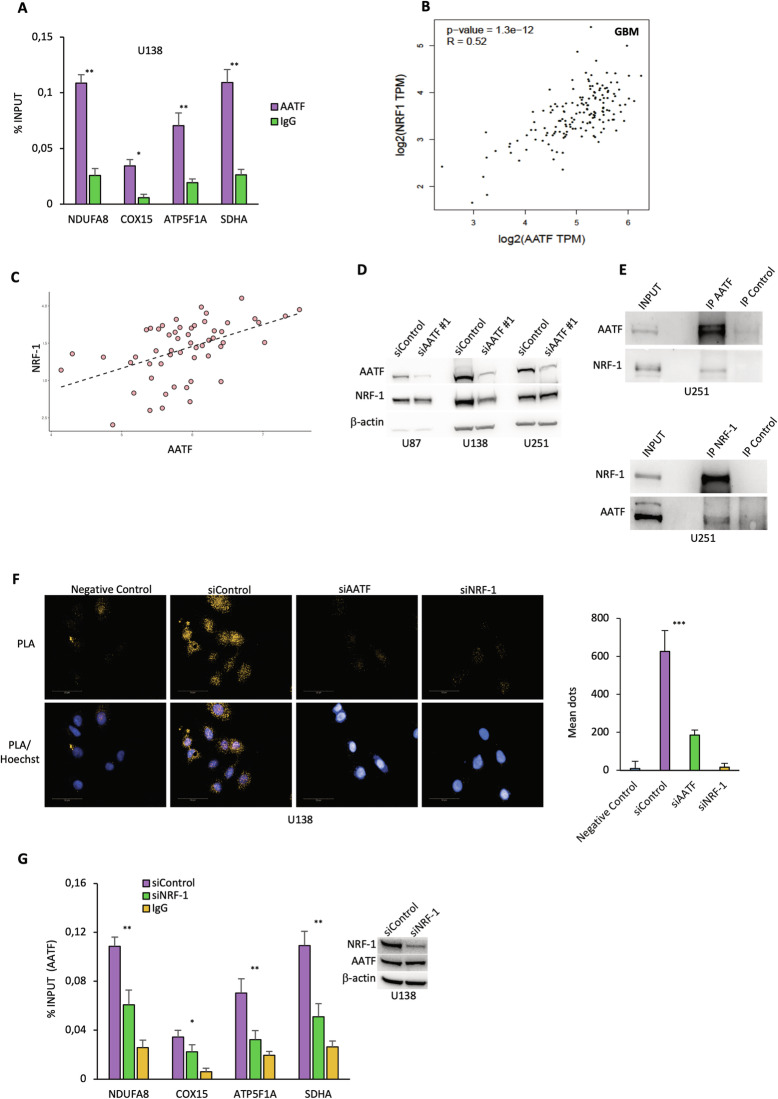
AATF binds to the OXPHOS gene promoters and promotes their transcription through NRF-1. **A** AATF binding on different OXPHOS gene promoters was evaluated by Chromatin Immunoprecipitation (ChIP) assays with a specific AATF antibody in U138 cells. The percentage of precipitated DNA was evaluated by qRT-PCR and calculated based on the ChIP input. **B** Correlation analysis of AATF and NRF-1 expression in GBM patients using the GEPIA tool. **C** Scatterplot representing the relationship between AATF and NRF-1 expression across Glioblastoma cell lines derived from the DepMap portal. The dashed line indicates a positive correlation (Pearson correlation = 0.49) between the expression of the two genes. **D** Representative WB of total cellular extracts from different GBM cell lines depleted or not for AATF expression. β-actin was used as loading control. **E** Nuclear extracts from U251 cells were subjected to immunoprecipitation with either AATF (top) or NRF-1 (bottom) antibodies. Immuno-precipitated complexes were then analysed by WB with the indicated antibodies. Input corresponds to 10% of the total extract used for immunoprecipitation. **F** Representative images showing the interaction between AATF and NRF-1 revealed by PLA in U138 cells depleted or not for AATF or NRF-1 expression. Negative control was performed by omitting NRF-1 primary antibody, whilst nuclei were stained with Hoechst dye. **G** AATF occupancy at OXPHOS gene promoters was evaluated by ChIP assay with a specific AATF antibody in U138 cells depleted or not for NRF-1 expression. The amount of precipitated DNA was evaluated by qRT-PCR and calculated based on the ChIP input. Data are presented as the mean ± SD of three independent experiments. Western blot shows the transfection efficiency of the experiment previously described. *p ≤ 0.05, **p ≤ 0.01, ***p ≤ 0.001, n.s. not significant.

Nuclear Respiratory Factor 1 (NRF-1) is a transcriptional factor mainly involved in controlling the expression of genes encoding the respiratory chain proteins [62, 63]. A previous analysis performed in our laboratory revealed that AATF could be found on chromatin regions enriched for binding motifs of NRF-1 [46]. Based on this evidence, we hypothesised that AATF could regulate the transcription of the OXPHOS genes by binding their promoter at the NRF-1 binding sites. Therefore, we carried out Chromatin Immunoprecipitation (ChIP) experiments in U138 cells with a specific antibody against AATF. Quantitative real-time analysis of the immunoprecipitated DNA using primer pair sets spanning the promoter region containing the binding motif for NRF-1 of selected OXPHOS genes, enabled us to demonstrate that AATF indeed regulate their expression by binding these promoter regions. The specificity of this association was confirmed with control ChIP with a non-specific antibody that did not show any significant enrichment (Fig. 5A). Notably, the expression of NRF-1, similarly to that of AATF, has been associated with progression and prognosis of GBM [27, 28]. Interestingly, a correlation analysis of the expression of AATF and NRF-1 on GBM patients revealed a positive (R: 0.52) and significant (*P*-value = 1.3e-12) correlation between these two proteins (Fig. 5B). This evidence was also confirmed in GBM cell lines as revealed by analysis of data from DepMap portal (Fig. 5C). To further investigate the relationship between AATF and NRF-1, we analysed NRF-1 expression levels in several GBM cell lines depleted or not for AATF expression. As shown in Fig. 5D, in some cases, AATF depletion was associated with a decrease in NRF-1 protein levels. To clarify whether this downregulation was exerted at transcriptional or translational level, NRF-1 transcript levels were analysed in AATF-depleted GBM cells. Interestingly, AATF does not significantly affect NRF-1 transcription (Fig. S7A), however, treatment with the proteasome inhibitor MG132 partly rescued the AATF-induced down-regulation of NRF-1 suggesting that AATF may control NRF-1 expression level through a post-translational mechanism involving proteasome degradation (Fig. S7B). Moreover, subcellular fractionation showed that AATF silencing also leads to a redistribution of NRF-1 from the nuclear to the cytoplasmic compartment (Fig. S7C). To gain further insight into the AATF/NRF-1interplay, we investigate whether the two proteins were able to interact. To this aim, we performed co-immunoprecipitation experiments with total cell extracts of U251 and U138 cells. As shown in Figs. 5E and S7D, NRF-1 was detected in AATF precipitate, thus indicating an interaction between the two proteins. Moreover, this interaction was also confirmed in reciprocal co-immunoprecipitation experiments (Figs. 5E and S7D), and by PLA, in which protein-protein interactions are visualised as discrete spots by fluorescence microscopy (Figs. 5F and S7E). Therefore, according to these results, we hypothesised that NRF-1 could mediate AATF association to the promoter region of the OXPHOS genes. To confirm this hypothesis, we performed ChIP experiments using anti-AATF antibody in U138 cells depleted or not for NRF-1 expression. As shown in Fig. 5G, NRF-1 depletion induces an important reduction in the amount of AATF on the promoters of OXPHOS genes. Altogether, these results reveal that AATF binding to OXPHOS gene promoters is mediated by NRF-1, showing a functional relationship between these two proteins.

### AATF is required for NRF-1 -dependent OXPHOS gene transcription

**Fig. 6:**
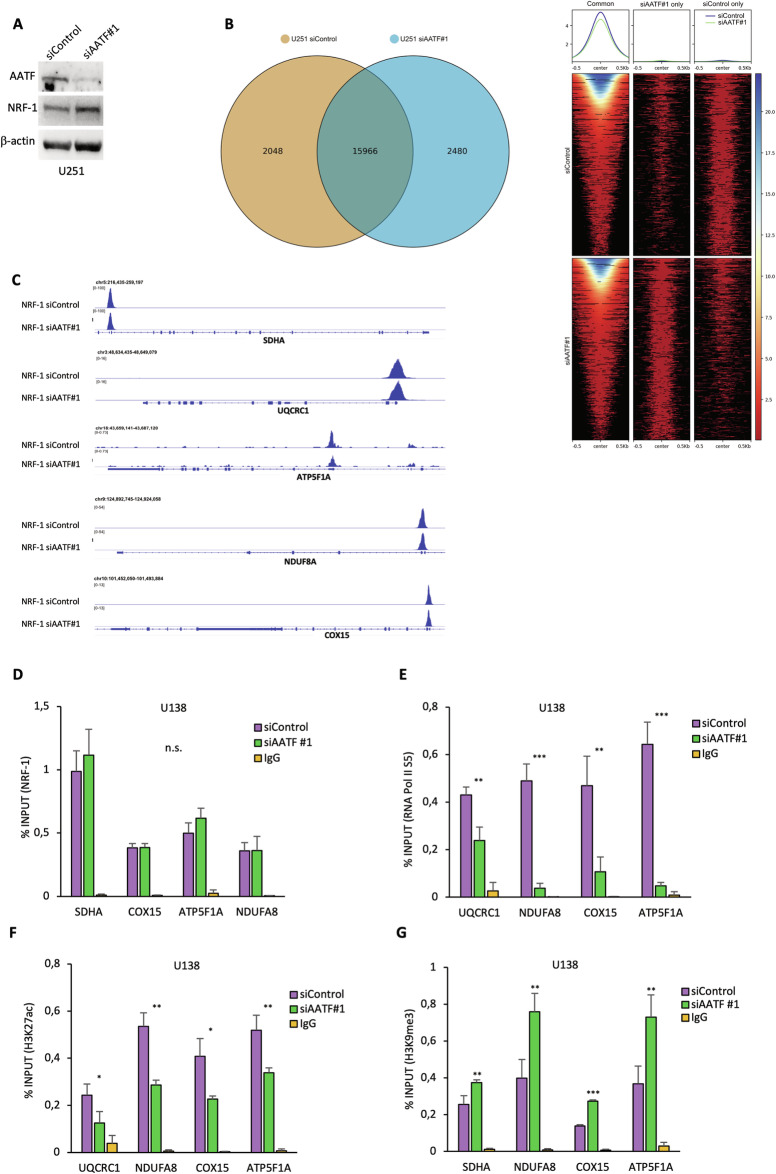
AATF is required for NRF-1- dependent OXPHOS gene transcription. **A** WB with the indicated antibodies of U251 cells transiently transfected with siControl or siAATF #1 oligonucleotides and subjected to ChIP-seq using anti-NRF-1 antibody. **B** Left: Venn diagram representing the intersection of peaks detected in NRF-1 ChIP-seq of siControl and siAATF cells. Right: NRF-1 ChIP-seq signal intensity of common and condition-specific peaks in siControl and siAATF cells. **C** NRF-1 ChIP-seq signal in siControl and siAATF #1 samples at SDHA, UQCRC1, ATP5F1A, NDUFA8, COX15 loci. **D** U138 cells transiently transfected with siControl or siAATF #1 oligonucleotides for 72 h were subjected to ChIP assay with a specific NRF-1 antibody. The amount of precipitated DNA was evaluated by qRT-PCR and calculated based on the ChIP input. **E** Pol II S5 occupancy at OXPHOS gene promoters was evaluated by ChIP assay in U138 cells depleted or not for AATF expression as described in (**D**). **F**, **G** U138 cells treated as in (**D**) were subjected to ChIP assay with specific antibodies for H3K27ac (Histone H3 acetylated at lysine 27) or H3K9me3 (Histone H3 trimethylated at lysine 9). Data are presented as the mean ± SD of three independent experiments. *p ≤ 0.05, **p ≤ 0.01, ***p ≤ 0.001, n.s. not significant.

To further elucidate the molecular mechanism through which AATF regulates the NRF1-dependent transcription of the OXPHOS genes, we evaluated whether it could also affect NRF-1 occupancy on their promoters. To this aim, we performed ChIP-seq experiments in U251 cells depleted or not for AATF expression, using a specific NRF-1 antibody. As shown in Fig. 6B, AATF downregulation does not completely impair NRF-1 binding to most of its specific recognition motifs, suggesting that while NRF-1 recruitment to OXPHOS gene promoters is necessary, it is not sufficient for transcriptional activation in the absence of AATF (Fig. 6A, B, C). Since AATF can regulate transcription by modulating RNA Polymerase II recruitment onto DNA as well as chromatin accessibility [29, 46], we verified whether it could affect these events also in this circumstance. To this aim, we performed ChIP experiments in GBM cells depleted or not for AATF expression, with antibodies against NRF-1 and RNA Pol II phosphorylated at ser 5 (Pol II S5), which specifically localises at promoter regions. As shown in Fig. 6D, E, while NRF-1 binding to the promoter of four selected OXPHOS genes does not change, RNA polymerase II S5 was recruited to a significantly lesser extent in AATF depleted cells. Consistent with these results co-immunoprecipitation experiments revealed that binding of RNA pol II S5 to NRF-1 decreases upon AATF silencing (Fig. S8A). Furthermore, analysis of the chromatin accessibility of these promoters showed a decrease in the acetylation levels of H3K27 (Fig. 6F), a histone modification commonly associated with a more relaxed chromatin [64], and a concomitant increase in the methylation levels of H3K9me3 (Fig. 6G), a marker of heterochromatin [65], upon AATF depletion. Furthermore, in line with previous reports [46], AATF depletion induced a global histone 3 acetylation reduction with a concomitant increase in H3K27 methylation levels (Fig. S8B). Overall, these findings indicate that AATF regulates OXPHOS gene transcription by affecting the recruitment of RNA polymerase II and by modifying chromatin accessibility at NRF-1 binding sites.

## Discussion

Metabolic reprogramming has been recognised as a key feature of malignant tumours. However, even if most cancer cells exhibit enhanced aerobic glycolysis to support their rapid growth and survival, an increasing number of studies is now showing that many tumours prioritise mitochondrial respiration as primary energy source [7, 8]. Consistently, upregulation of genes responsible for the OXPHOS pathway has been observed in different types of cancers and it has been associated with treatment resistance and poorer survival [3, 4]. In the present study, we explore the role of the transcriptional cofactor AATF in the regulation of proliferation and OXPHOS gene expression in GBM. Although a previous report has highlighted an upregulation of this protein in GMB tumour compared to normal tissue [50], its specific functions have only been partially identified. Here, we provide evidence that AATF is not only upregulated in GBM, but its high expression is also associated with adverse prognosis. Notably, we demonstrate that AATF downregulation induces cell cycle arrest of GBM cells, coupled with a decreased expression of the OXPHOS components as well as decreased mitochondrial respiration. Moreover, AATF-depleted cells showed a marked reduction in their ability to form colonies, in vitro and to induce tumour formation in vivo. Mechanistically, we found that AATF binds the promoters of the OXPHOS genes through a specific interaction with the transcription factor NRF-1 and promotes transcription by modulating their epigenetic state as well as RNA polymerase II recruitment. AATF is known to modulate transcription by interacting with specific transcription factors [35]. Here we identified this protein as a novel binding partner of NRF-1, with a fundamental role in NRF-1- dependent transcription of the OXPHOS genes. NRF-1 is a master regulator of mitochondrial functions regulating transcription of many nuclear-encoded genes required for mitochondrial biogenesis and respiratory functions, including mitochondrial transcription factor A (TFAM), which regulates mitochondrial DNA replication and transcription [66]. Interestingly, ChIP-seq data produced using a specific anti-AATF antibody revealed a strong association of AATF with TFAM promoter at NRF-1 binding motif, suggesting a much broader role for AATF/NRF-1 interaction in the regulation of mitochondrial activities [46]. Further experiments are surely needed to better investigate this interaction in the setting of GBM and to explore its functional roles. Indeed, since both AATF and NRF-1 participates in many cellular pathways involved in proliferation and survival, it will be interesting to verify whether they may cooperate to integrate mitochondrial functions to other pathways involved in GBM development and progression. Notably, the complex AATF/NRF-1 can be surprisingly detected not only in the nucleus but also in the cytoplasm (Fig. 5F), suggesting the existence of a more complex relationship between the two proteins. Indeed, we provide evidence that AATF could modulate NRF-1 stability and subcellular localisation, affecting at least in part NRF-1 presence onto the DNA. However, the mechanisms underlying these phenomena have not been clarified and need additional investigation. Hence, in-depth characterisation of AATF/NRF-1 interaction, including mapping the protein domains responsible for the binding and identifying the post-translational modifications driving it, will help to elucidate the functional impact of this complex in GBM tumorigenesis and could provide important insights for the design of targeted strategies for its inhibition. Consistent with the ability of AATF to induce transcriptionally active chromatin [46], we demonstrate that its depletion modulates the epigenetic state at the NRF-1 binding sites of OXPHOS gene promoters. The molecular mechanisms underlying this phenomenon are not yet clear and require further investigation. Since AATF depletion is associated with a global reduction in histone H3 acetylation levels, it is likely that it may involve Class I Histone Deacetylase recruitment, as demonstrated in other contexts [29, 34, 36, 46]. However, other possibilities cannot be excluded. First, AATF is part of complexes containing acetyltransferases [67], thus it could modulate chromatin accessibility by affecting also this class of enzymes. Moreover, the reduction in ATP levels observed in AATF-depleted cells as well as the possible alteration in NAD^+^/NADH ratio due to OXPHOS inhibition, could affect the activity of ATP-dependent chromatin remodelling enzymes and sirtuins, respectively. One of the main challenges in the management of GBM is its resistance to treatments. A recent study by Mi et al., demonstrates that AATF contributes to chemoradiotherapy resistance of glioblastoma stem cells by promoting efficient DNA repair [53]. In line with this report, we provide evidence that AATF depletion increases cell sensitivity to temozolomide, an alkylating agent currently used in GBM treatment. Furthermore, our RNA-seq enrichment analysis revealed a downregulation of the base excision repair pathway upon AATF depletion, suggesting that this protein may not only sustain cellular proliferation through OXPHOS regulation but also contributes to cell survival by modulating different pathways involved in the maintenance of genomic stability. In this context, it is worth to highlight that upregulation of OXPHOS pathways has also been linked to pharmacological resistance [7], hence, AATF may contribute to the maintenance of the resistant phenotype through this pathway as well. Although this study focused on the regulation of OXPHOS genes by AATF, RNA-seq analysis clearly revealed that its depletion triggers a complex cellular response involving pathways that directly affect DNA replication and genome integrity maintenance as well as anabolic metabolism. This evidence strongly supports AATF’s central role in GBM cell proliferation and suggests therapeutic approaches based on its downregulation. However, no compounds capable of inhibiting its activity have been identified to date, and the lack of a 3D structure makes their identification particularly challenging. Therefore, a detailed investigation of the molecular mechanisms underlying AATF upregulation during GBM progression will be essential to identify effective strategies for restoring its expression to physiological levels.

## Supplementary information

Supplementary information

Original western blots

## Data Availability

High Through- put Sequencing data (RNA-seq, ChIP-seq) generated in this work have been submitted to the National Cancer Center for Biotechnology Information (NCBI) Gene Expression Omnibus database and assigned the identifier numbers: GSE299968 (RNAseq) and GSE299967 (ChIPseq). All other data supporting the findings of this study are available from the corresponding authors on reasonable request.
