# Supplementary material for: AATF supports proliferation of glioblastoma cells by sustaining mitochondrial respiration through an NRF-1-dependent mechanism
Source: Cell Death Dis. 2026 Mar 24;17(1):349. doi: 10.1038/s41419-026-08617-0 (PMC13039372; doi:10.1038/s41419-026-08617-0)
Supplement: Supplementary file 1 — Supplementary information [file 41419_2026_8617_MOESM1_ESM.pdf]

SUPPLEMENTARY INFORMATION

AATF supports proliferation of glioblastoma cells by sustaining mitochondrial respiration through an NRF-1-dependent mechanism

Cristina Sorino[1✉], Stefano Di Giovenale[1,4], Francesca Romana Auciello[2], Claudio Pulito[2], Federica Lo Sardo[2], Stefano Scalera[3], Francesca De Nicola[1], Valeria Catena[1], Ludovica Ciuffreda[1], Italia Falcone[1], Brindusa Ana Maria Arteni[5], Stefano Giuliani[1], Bruno Amadio[1], Giovanni Blandino[2], Maurizio Fanciulli[1✉] and Simona Iezzi[1]

1- Gene Expression and Cancer Models Unit, Department of Research and Advanced Technologies, Translational Research Area, IRCCS Regina Elena National Cancer Institute, 00144, Rome, Italy.

2- Translational Oncology Research Unit, IRCCS, Regina Elena National Cancer Institute, 00144, Rome, Italy.

3- Biostatistics, Bioinformatics and Clinical Trial Center, IRCCS Regina Elena National Cancer Institute, 00144, Rome, Italy.

4- Department of Computer, Control and Management Engineering, Sapienza University of Rome, Rome, Italy.

5- Pathology Unit, Tissue Biobank, IRCCS Regina Elena National Cancer Institute, 00144, Rome, Italy.

✉ maurizio.fanciulli@ifo.it; cristina.sorino@ifo.it

Tables of contents

Supplementary Materials and Methods

Supplementary Table S1

Supplementary Table S2

Supplementary Figures 1-8

Supplementary Figure Legends 1-8

SUPPLEMENTARY MATERIALS AND METHODS

Apoptosis Evaluation

Apoptosis of cells was analysed by using the Annexin V-FITC Apoptosis Kit 300 (Thermo Fisher Scientific #BMS500FI-300) following manufacturer's instructions. Stained cells were analysed with a Guava easyCyte 8HT flow cytometer equipped with Guava Software 2.1 (Millipore) according to the manufacturer's instructions.

Viral production and dCas9-KRAB system development

Glycerol stock for the dCas9-BFP-KRAB plasmid was obtained from Addgene (plasmid catalog #46911). To produce lentiviral particles, DNA obtained with Nucleo Bond Xtra Midi (Macherey-Nagel) was transfected into human embryonic kidney 293 T (HEK293-T) cells with a pPACKH1 packaging plasmid mix (System Biosciences) and Lipofectamine 3000 (Thermo Fisher Scientific). Viral-containing supernatant was collected 48 and 72 hours after transfection and concentrated with PEG-it Virus Precipitation Solution (System Biosciences) for 18 hours at 4°C. Next, the sample was centrifuged at 1500 x *g* for 30 min and the final pellet containing virus particles was resuspended in phosphate buffered saline (PBS) and stored at -80 °C. U138 cells were infected with the dCas9-BFP-KRAB virus particles in the presence of 8 µg/ml polybrene (Santa Cruz Biotechnology) for 48 hours and then sorted with flow cytometry for stable BFP expression to obtain a pure population. Two specific sgRNAs targeting the AATF gene were custom designed using the CHOP CHOP algorithm: sgAATF#1: AATAAAGCATAGCGTAGCCG and sgAATF#2: CGAATCTCGCACAAATCTCG (GenScript Biotech, New Jersey, USA). sgRNA negative control (cat.n. A35526) was purchased by Thermo Fisher Scientific.

Subcellular Fractionation

For cytoplasmic and nuclear extract preparation cells were incubated in ice for 5 min in hypotonic buffer (50 mM TRIS pH 7.5, 10 mM NaCl, 5 mM ethylenediaminetetraacetic acid (EDTA), 0.05% NP40) supplemented with protease and phosphatase inhibitors (1 µg/ml aprotinin, 1 µg/ml leupeptin, 1 mM Na_3VO_4, 10 mM phenylmethylsulfonyl fluoride (PMSF)). Following low speed centrifugation, supernatant was collected as

cytoplasmic fraction. Nuclear pellet was washed in hypotonic buffer, and then re-suspended in buffer C (20 mM HEPES pH 7.9, 25% glycerol, 420 mM NaCl, 1.5 mM $MgCl_2$, 0.2 mM EDTA, protease and phosphatase inhibitors) and sonicated for 10 sec. Nuclear extracts were clarified by centrifugation at 12 000 rpm at 4°C for 15 min.

Measurement of intracellular ROS

Intracellular ROS levels were measured using the fluorescent dye 2′,7′-dichlorodihydrofluorescein diacetate (DCFDA) (Sigma-Aldrich). Cells depleted or not for AATF expression were seeded in a 96-well PhenoPlate (Perkin Elmer, USA) and cultured overnight. After this time, the cells were treated with 10 µM of DCFDA and incubated at 37 °C for 30 min. Next, the fluorescence intensity was measured at 488 nm excitation wavelength and 525 nm emission wavelength using Opera Phenix High-content System (Perkin Elmer, USA) and analysed with Harmony™ high content imaging and analysis software (Perkin Elmer, USA, version 4.8).

Computational methods

GlioVis methods

Clinical data from the REMBRANDT cohort were obtained through the GlioVis portal. To examine the distribution of AATF expression, patients were stratified by histology, tumor grade, and molecular subtype. For each analysis, samples lacking the relevant clinical annotation were excluded.

Venn diagram methods

To assess the modulation of OXPHOS signature in different cancer types depleted for AATF, we retrieved RNA-seq datasets for BIU-87 (GSE248071) and KMS27 (GSE178868). For BIU-87, raw counts were downloaded and analysed using the standard DESeq2 differential expression pipeline. For KMS27, we obtained the differential expression results directly from GEO. In all datasets, we defined up-regulated genes as those with q-value < 0.05 and $\log_2 FC > 0.7$, and down-regulated genes as those with q-value < 0.05 and $\log_2 FC < -0.7$. The analysis was performed through the venn.diagram function from VennDiagram R library (v1.7.3).

**Rabbit Polyclonal Antibodies**		
	**Source**	**Cat. Number**
**AATF**	Fanciulli et al., 2000	
**AATF/Che-1**	Bethyl	A301-032A
**Cyclin B1 (D5C10)**	Cell Signaling Tecnologies	#12231
**p21**	Abcam	ab188224
**Caspase-7**	Cell Signaling Tecnologies	#9492
**Caspase-3**	Cell Signaling Tecnologies	#9662
**PARP**	Cell Signaling Tecnologies	#9542
**Phospho-Akt (Ser473)**	Cell Signaling Tecnologies	#9271
**Akt**	Cell Signaling Tecnologies	#9272
**Phospho-p44/42 MAPK (Erk1/2) (Thr202/Tyr204)**	Cell Signaling Tecnologies	#4370
**p44/42 MAPK (Erk1/2)**	Cell Signaling Tecnologies	#9102
**RNA polymerase II CTD repeat YSPTSPS (phospho S5)**	Abcam	ab5131
**Acetyl-histone H3**	Merck Millipore	06-599
**H3K27me3**	Diagenote	C15410195
**Histone H3**	Abcam	ab18521
**Mouse Monoclonal Antibodies**		
	**Source**	**Cat. Number**
**NRF1**	Diagenote	sc-28714
**anti-total OXPHOS cocktail**	Abcam	ab110411
**α - tubulin**	Calbiochem	CP06
**β - actin**	Sigma Aldrich	A5441
**PCNA**	Santa Cruz Biotechnology	sc-25280

Table S1. Antibodies used in this study.

Real Time PCR Oligos		
**SDHA**	Forward	5' - CAGCATGTGTTACCAAGCTGT - 3'
	Reverse	5’ - GGTGTCGTAGAAATGCCACCT - 3'
**NDUFA8**	Forward	5' - CCCAACAAGGAGTTTATGCTCT - 3'
	Reverse	5' - CACAGTGACGTTTTATCTGCCT - 3’
**COX15**	Forward	5' - AGTCTGGCCTCTCGATGGTAG - 3'
	Reverse	5' - ACCCCACATTCGGTGTGAGTA - 3'
**UQCRC1**	Forward	5' - GGGGCACAAGTGCTATTGC - 3'
	Reverse	5' - GTTGTCCAGCAGGCTAACC - 3'
**ATP5F1A**	Forward	5' - CCGGAATTCGGATAAGACCAAACTGGCT - 3’
	Reverse	5' - CCGCTCGAGGAGTTCTCGAAGGAGCTG - 3'
**β-actin**	Forward	5' - GACAGGATGCAGAAGGAGATTACT - 3'
	Reverse	5' - TGATCCACATCTGCTGGAAGGT - 3'
**AATF**	Forward	5' - CCGGAATTCGGATAAGACCAAACTGGCT - 3’
	Reverse	5' - CCGCTCGAGGAGTTCTCGAAGGAGCTG - 3'
**NRF-1**	Forward	5' - GCTGATGAAGACTCGCCTTCT - 3’
	Reverse	5' - TACATGAGGCCGTTTCCGTTT - 3'
**MFN1**	Forward	5’ - CGGAACTTGATCGAATAGCC - 3’
	Reverse	5’ - AGAGCTCTTCCCACTGCTTG - 3’
**MFN2**	Forward	5’ - ATGCATCCCCACTTAAGCAC - 3’
	Reverse	5’ - AGCACCTCACTGATGCCTCT - 3’
**FIS1**	Forward	5’ - AGCTGGTGTCTGTGGAGGAC - 3’
	Reverse	5’ - ACGATGCCTTTACGGATGTC - 3’
**DNM1L**	Forward	5’ - AGATCTCATCCCGCTGGTC - 3’
	Reverse	5’ - CAGATCCTCGAGGCAAGAAG - 3’
**MFF**	Forward	5’ - AAACGCTGACCTGGAACAAG - 3’
	Reverse	5’ - TTTTCAGTGCCAGGGGTTTA - 3’
**ChIP Oligos**		
**NDUFA8**	Forward	5' - AAGTTTGAGGATTAAGCGACA - 3'
	Reverse	5' - GGTCCCCTCCGCAGCTTTGGG - 3'
**COX15**	Forward	5' - TCTAGGCGCTGCCCTAGGAGC - 3'
	Reverse	5' - AACGCAGGCGCACTTCTGTTC - 3
**ATP5F1A**	Forward	5' - GCAGTTTTCTGACCTTCAGCG - 3'
	Reverse	5' - AGAAGGGGGATCTTGGGTGA - 3'
**SDHA**	Forward	5' - TAAACAAGAGGTCGGAGGC - 3'
	Reverse	5' - GGGCACCAACATTTTTAAAGC - 3'
**UQCRC1**	Forward	5' - AAGGCTGGCGTCCGCAGCAGG - 3'
	Reverse	5' - CCACTGCTAACAGCCCACACC - 3’

Table S2. Oligo sequence used in this study.

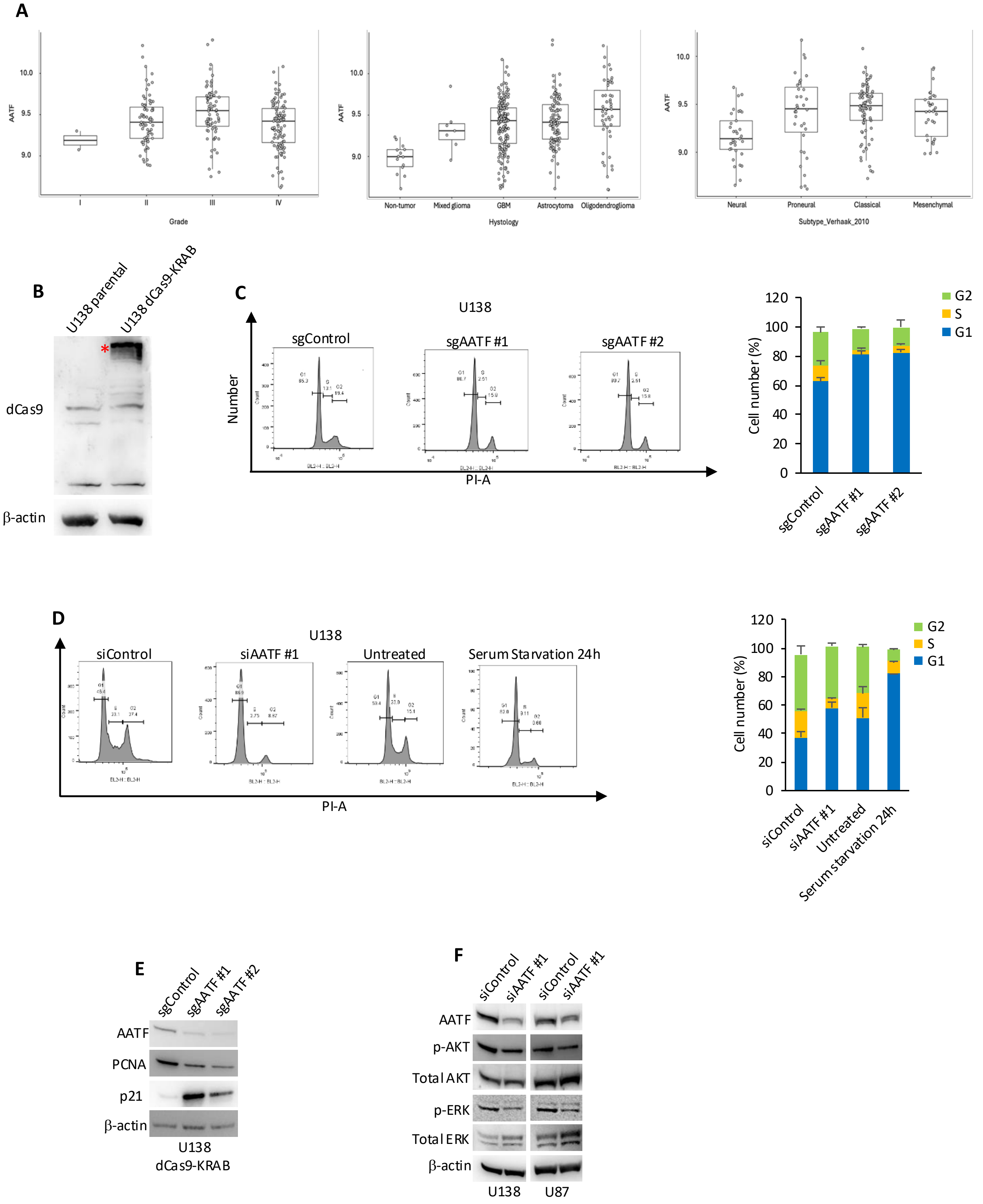

Supplementary Figure 1

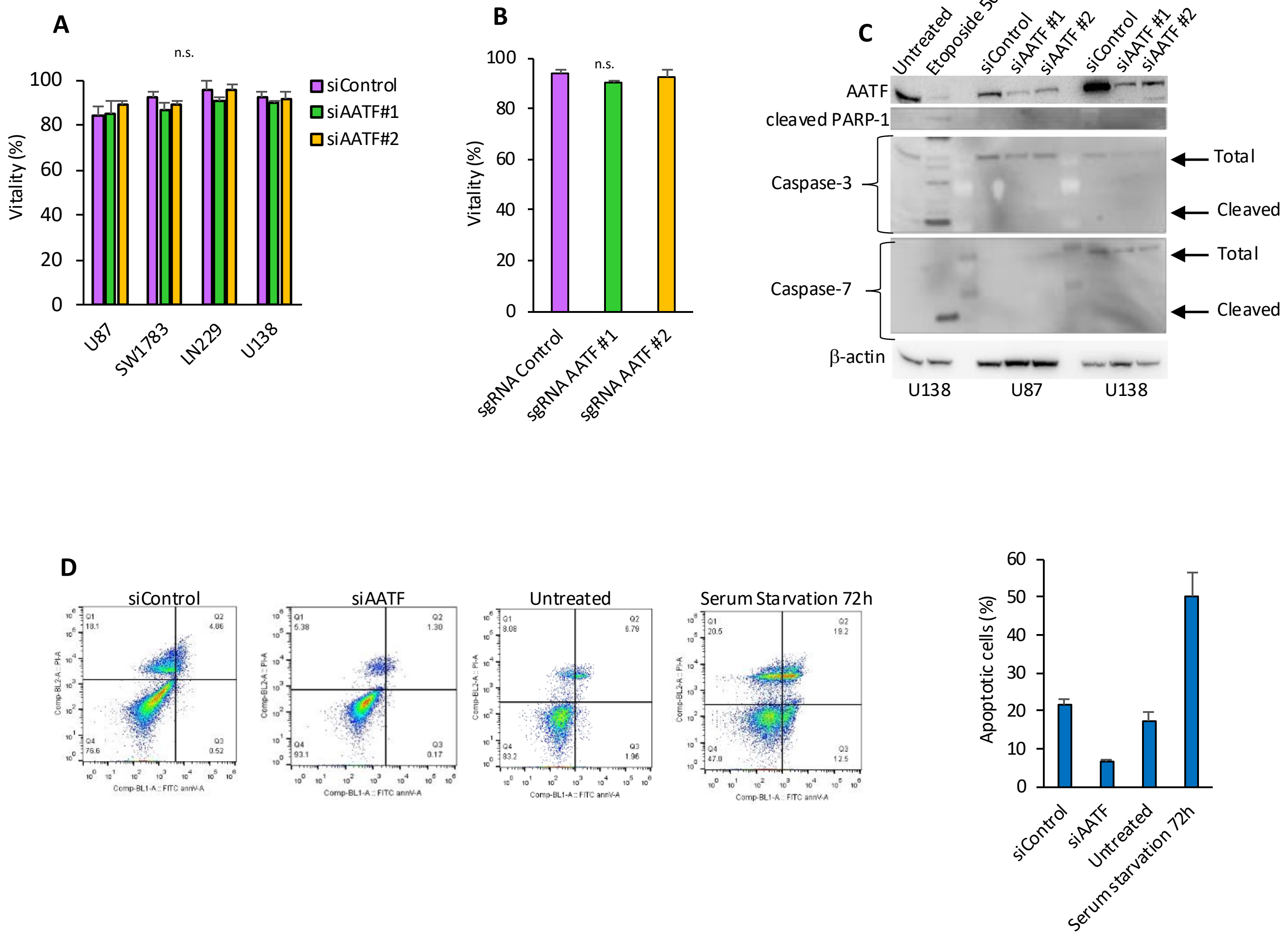

Supplementary Figure 2

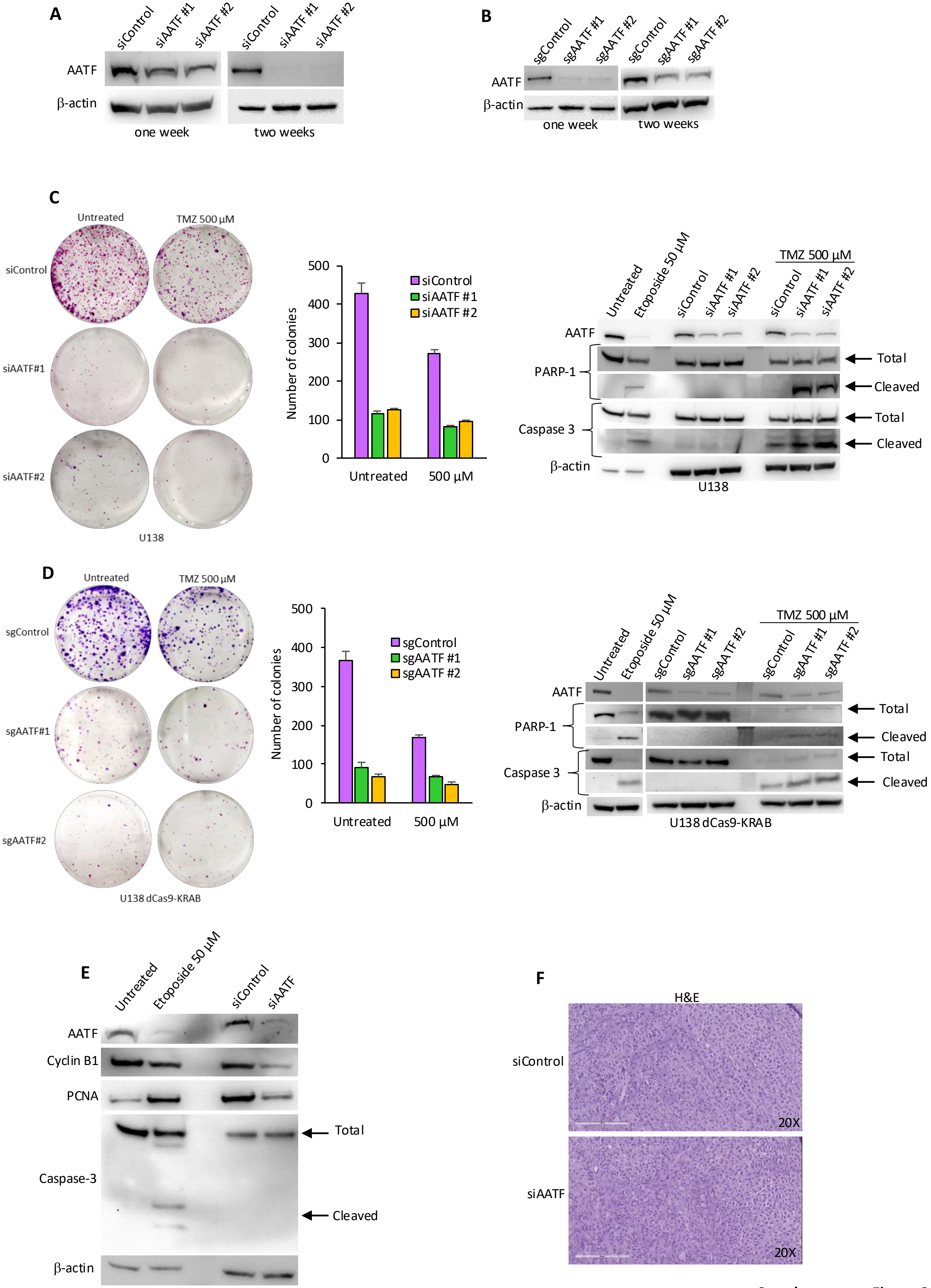

Supplementary Figure 3

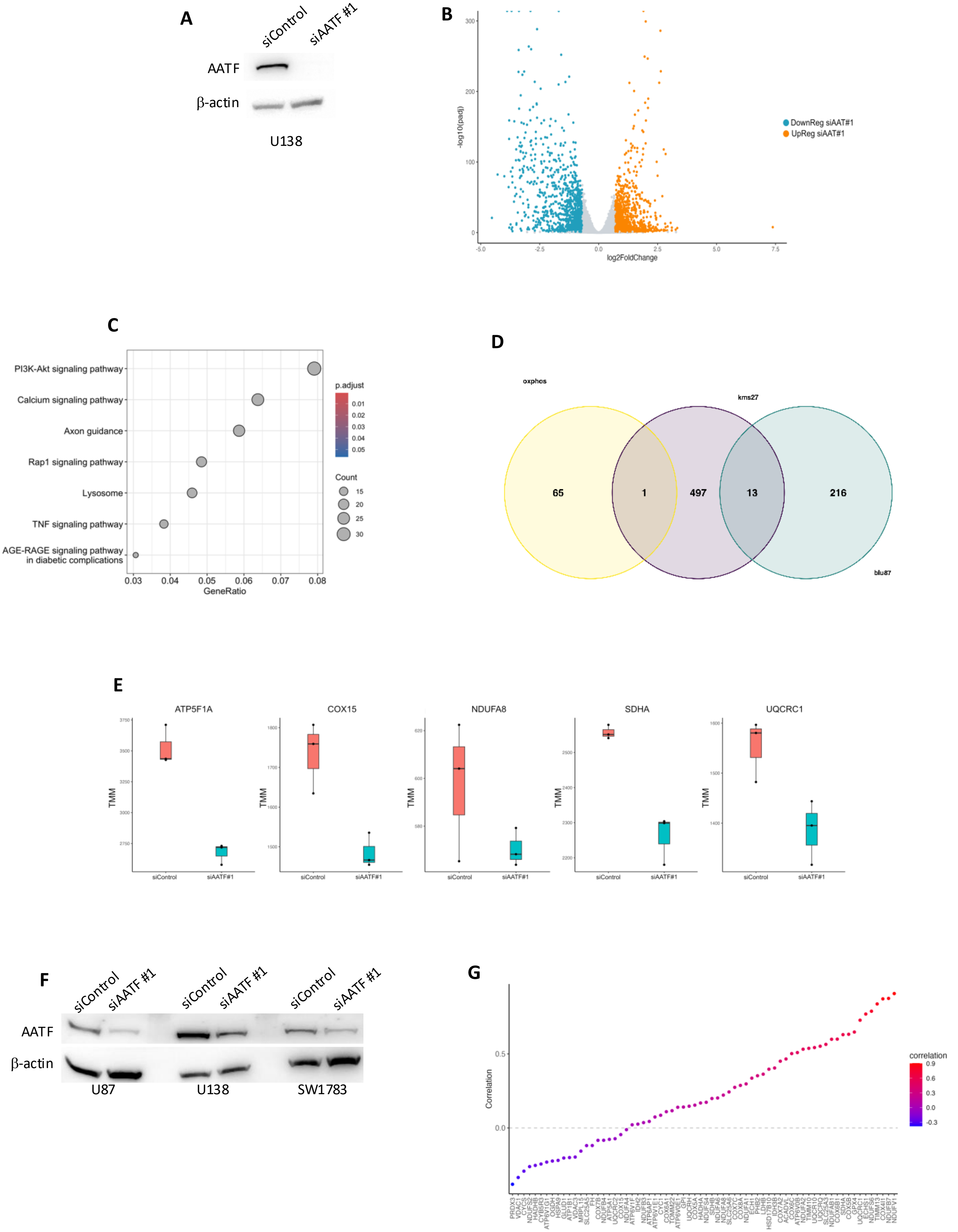

Supplementary Figure 4

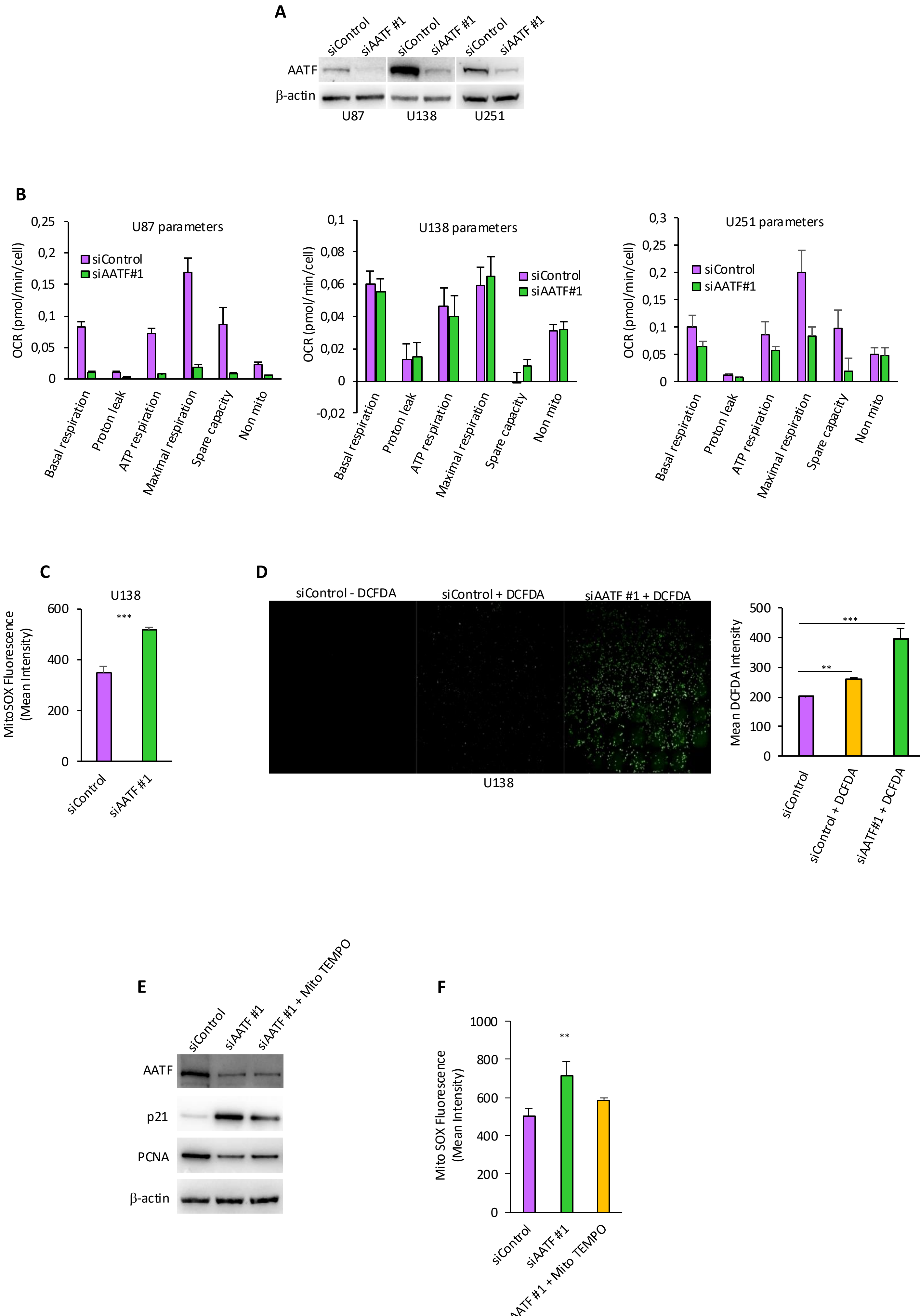

Supplementary Figure 5

A

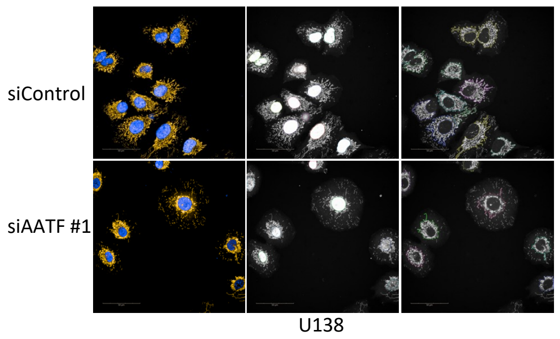

B

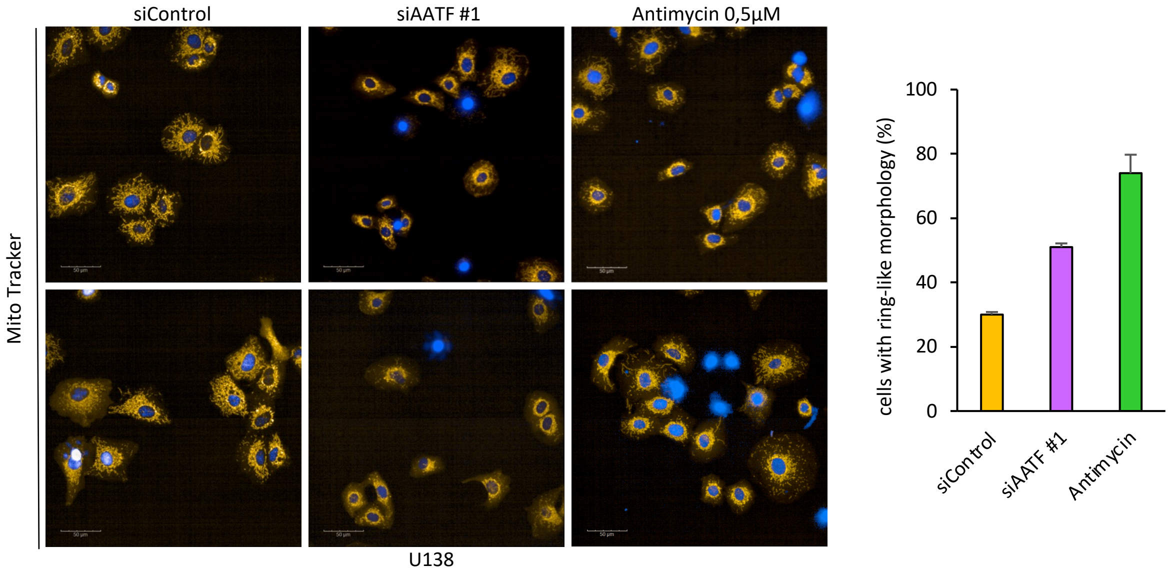

C

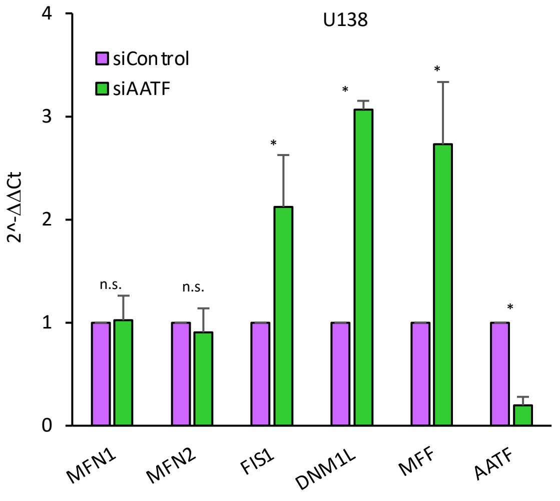

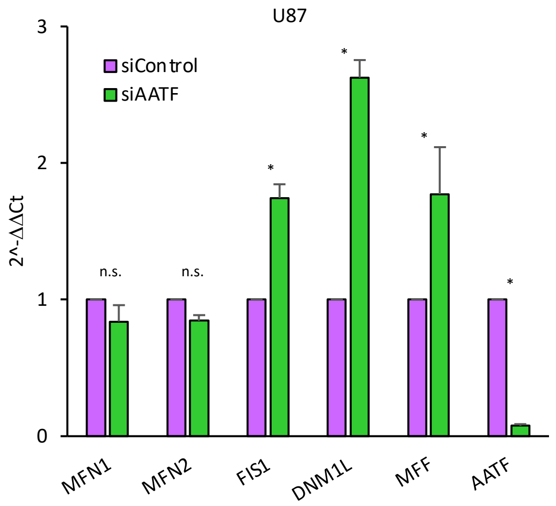

Supplementary Figure 6

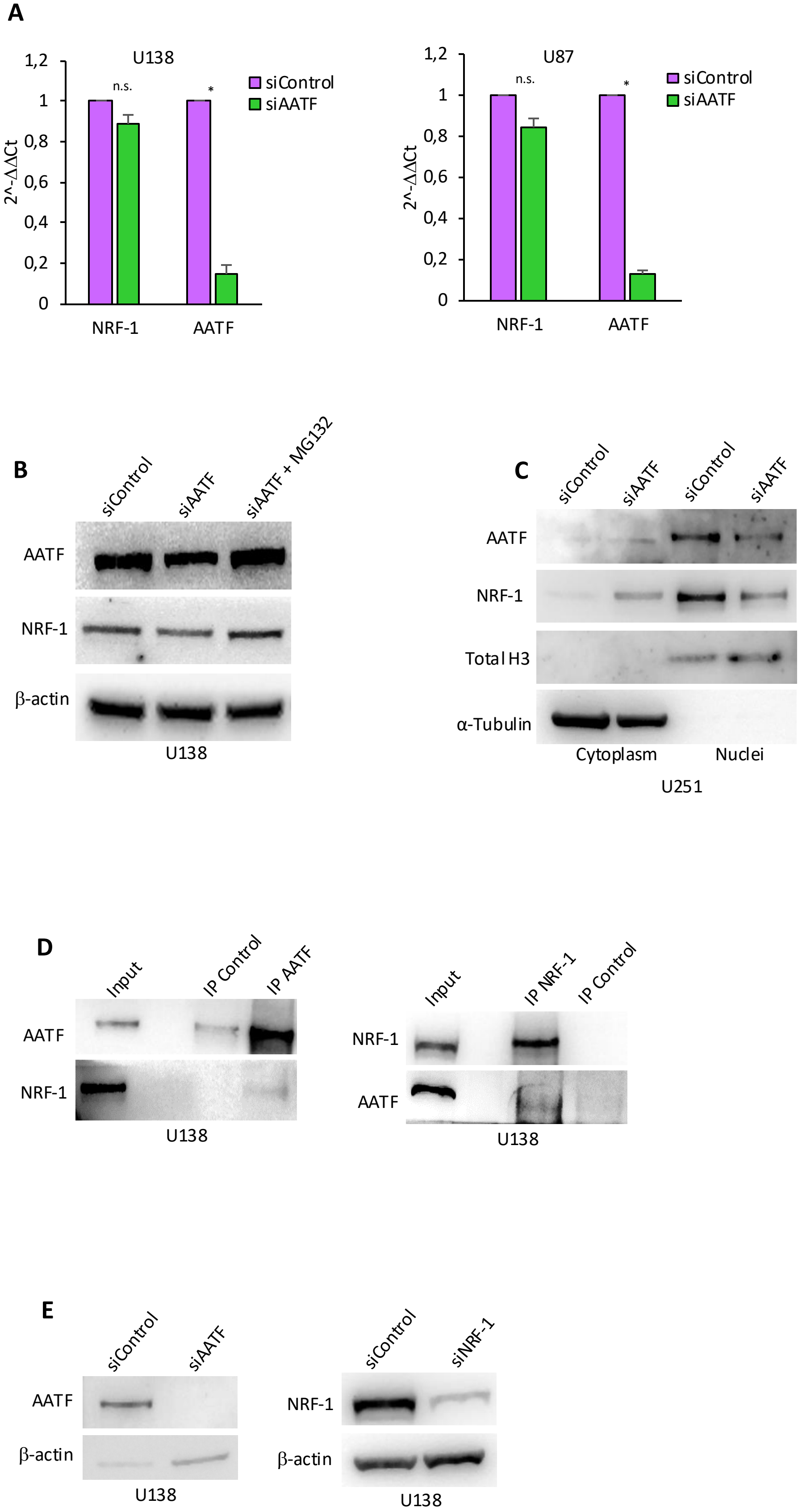

Supplementary Figure 7

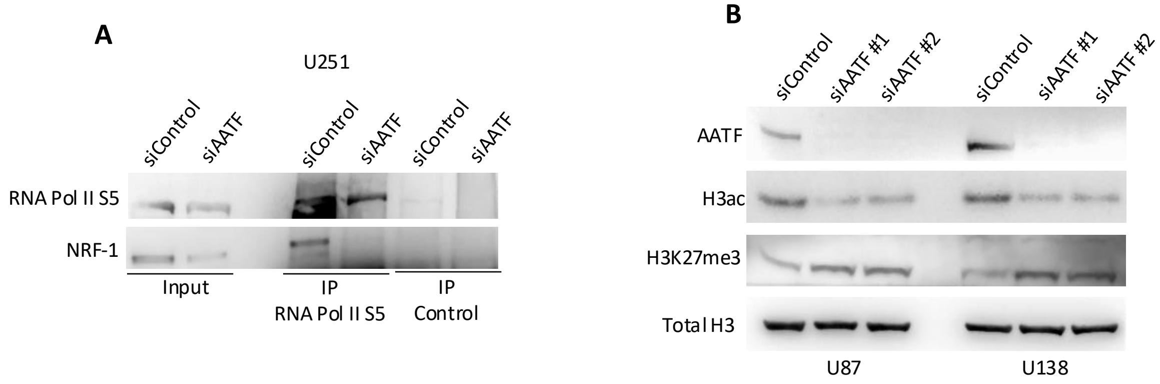

Supplementary Figure 8

SUPPLEMENTARY FIGURE LEGENDS

Supplementary Fig. S1 (related to Figure 1). AATF promotes proliferation of GBM cells, in *vitro*. (A) Boxplots illustrating the distribution of AATF transcript levels in GlioVis patients, categorized by histology, grade, and subtype. Each point represents an individual sample. **(B)** WB analysis with the indicated antibodies of parental or stably expressing the dCas9-KRAB transcriptional repressor complex U138 cells. **(C)** Flow cytometry analysis of cell cycle distribution of U138 cells stably expressing the dCas9-KRAB transcriptional repressor complex and transfected with Control or AATF targeting sgRNAs (sgAATF#1 and sgAATF#2) (left panels). Percentages of cells in each phase of the cell cycle were measured by FlowJo software (right panel). **(D)** Flow cytometry analysis of cell cycle distribution of U138 cells depleted or not for AATF expression or cultured or not for 24 hours in serum-free media. Percentages of cells in each phase of the cell cycle were measured by FlowJo software. Data are presented as the mean ± SD of three independent experiments. *p≤0,05, **p≤0,01, ***p≤0,001, n.s. = not significant. **(E)** Representative WB analysis of dCas9-KRAB U138 cells treated as in C assessing the expression levels of the indicated proteins. **(F)** Representative WB analysis with the indicated antibodies of U138 and U87 cells transfected with siControl or siAATF oligonucleotides for 72 hours. β-actin was used as a loading control.

Supplementary Fig. S2 (related to Figure 1). AATF promotes proliferation of GBM cells, in *vitro*. (A) Viability assay performed by Trypan Blue exclusion method in the indicated cell lines transfected with two different siRNA oligonucleotides targeting AATF (siAATF #1-siAATF #2) or a control sequence (siControl) for 72 hours. **(B)** Viability assay performed by Trypan Blue exclusion method in U138 cells stably expressing the dCas9-KRAB transcriptional repressor complex and transfected with Control or AATF targeting sgRNAs (sgAATF#1 and sgAATF#2). **(C)** Representative WB with the indicated antibodies of U138 and U87 cells treated as described in A. Cells treated for 24 hours with etoposide 50 μM were used as a positive control for apoptosis induction. β-actin was used as a loading control. **(D)** Flow cytometry analysis of Annexin-V-FITC and PI stained U138 cells treated as in A and cells cultured or not for 72 hours in serum-free media. Data are presented as the mean ± SD of three independent experiments. *p≤0,05, **p≤0,01, ***p≤0,001, n.s. = not significant.

Supplementary Fig. S3 (related to Figure 2). AATF promotes proliferation of GBM cells, in *vivo*. (A-B) Representative WB analysis of U138 cells transfected with control siRNA (siControl) or two distinct siRNAs targeting AATF (siAATF #1 and siAATF #2) (A) or of dCas9-KRAB U138 cells transfected with Control or AATF targeting sgRNAs (sgAATF#1 and sgAATF#2) (B) and analysed by colony formation assay as described in Figs. 2A and 2B. β-actin was used as a loading control. **(C-D)** Left: representative plates of clonogenic assay performed on U138 (C) or U138 dCas9-KRAB cells (D) treated as in A or B. Cells were harvested 72 hours after transfection, counted, and re-plated at low density to allow colony formation in presence or absence of 500 µM Temozolomide (TMZ) for one week. Colonies were then fixed and stained with crystal violet. Middle: bar plots showing the quantification of clonogenic assays performed with ImageJ software. Right: representative WB with the indicated antibodies of the U138 cells used in the clonogenic assays. Etoposide-treated cells were included as a positive control for apoptosis induction. β-actin was used as a loading control. **(E)** WB analysis of U138 cells transfected with siRNA targeting AATF or a control sequence and subcutaneously injected in nude mice. Etoposide-treated cells were included as a positive control for apoptosis induction. **(F)** Representative IHC images of hematoxylin and eosin (H&E) staining of the same tumor tissue showed in Fig. 2F and captured at 20× magnification using Aperio Image Scope system equipped with a Digital Image Capture software. Data are presented as the mean ± SD of three independent experiments. *$p \leq 0,05$, **$p \leq 0,01$, ***$p \leq 0,001$, n.s. = not significant.

Supplementary Fig. S4 (related to Figure 3). AATF regulates OXPHOS gene expression in GBM cells. (A) Representative WB analysis with the indicated antibodies in U138 cells transiently transfected with siControl or siAATF #1 oligonucleotide and subjected to RNA-seq. **(B)** Volcano plot showing differentially expressed genes between siControl and siAATF#1 conditions in U138 cells. Each point represents a gene, with the x-axis indicating the log2 fold change and the y-axis representing the log10(q value). Differentially expressed genes are coloured blue for down-regulated genes and orange for up-regulated genes. **(C)** The scatterplot represents the ontologies enrichment analysis of siAATF up-regulated genes. The x-axis represents the GeneRatio (the proportion of input genes associated with each GO term), dot size reflects the number of genes contributing to the term, and dot colour indicates the adjusted p-value (p.adjust). **(D)** Venn diagram

showing the overlap between the OXPHOS gene signature and genes modulated upon AATF depletion in the KMS27 and BIU87 cell lines. **(E)** Boxplot depicting the expression levels (TMM) of five mitochondrial genes (ATP5F1A, COX15, NDUFA8, SDHA and UQCRC1) in siControl and siAATF#1 cells. Each boxplot displays the median, interquartile range, and data points for each condition. **(F)** Representative WB analysis with the indicated antibodies of different GBM cell lines transfected with siControl or siRNA oligonucleotides and subjected to qRT-PCR analysis of the OXPHOS genes. **(G)** Scatter plot representing the Pearson correlation between AATF and the OXPHOS genes expression in the CGGA dataset of 1019 patients affected by Glioblastoma. Genes are ordered along the X-axis by increasing correlation. Point colors indicate the strength and direction of the correlation. The dashed line marks the zero correlation, and it divides positive from negative correlation.

Supplementary Fig. S5 (related to Figure 4). AATF sustains mitochondrial respiration in GBM cells. (A) Representative WB analysis with the indicated antibodies of different GBM cell lines transiently transfected with siControl or siAATF #1 oligonucleotides and subjected to oxygen consumption rate evaluation (OCR). **(B)** Respiratory parameters as determined by OCR relative to GBM cell lines treated as in A. **(C)** Bar plots showing the mean of MitoSOX fluorescence intensity calculated with automated Harmony software of the experiment described in Figure 4C. **(D)** Intracellular ROS levels were measured by DCFDA assay in U138 cells treated as in A. Left: representative fluorescence images, in which the intensity of the green fluorescence indicates ROS concentration, acquired with confocal Opera Phenix High Content Screening System using a 40x air objective. Right: Bar plots showing the mean fluorescence intensity calculated with automated Harmony software. **(E)** WB analysis with the indicated antibodies of the protein extracts from the cells used in the experiment described in figure 4E. **(F)** Bar plots showing the mean of MitoSOX fluorescence intensity calculated with automated Harmony software of the experiment described in Figure 4E. Data are presented as the mean ± SD of three independent experiments. *p≤0,05, **p≤0,01, ***p≤0,001, n.s. = not significant.

Supplementary Fig. S6 (related to Figure 4). AATF sustains mitochondrial respiration in GBM cells. (A) Representative confocal images of U138 cells transiently transfected with siControl or siAATF #1 oligonucleotides and stained with Mito Tracker Red CMX Ros (mitochondria labelling) and Hoechst dye

(nuclei staining). Automated confocal microscopy was performed on Opera Phenix High–Content Screening System using a 63× objective. The left column displays merged images of mitochondrial (yellow) and nuclear (blue) staining. The right panels show segmentation masks used for computational analysis. In particular, the middle panels show the nuclear segmentation masks, while the rightmost panels display the segmentation of the mitochondrial network. Scale bars: 50 µm. **(B)** Left: representative confocal images of U138 cells treated as in A and stained with Mito Tracker Red CMX Ros (mitochondria labelling) and Hoechst dye (nuclei staining). Automated confocal microscopy was performed on Opera Phenix High–Content Screening System using a 63× objective. Treatment with the mitochondrial toxin antimycin 0.5 µM for 5 minutes was used to induce mitochondrial fragmentation. Right: bar plots showing the percentage of cells with a ring-like mitochondrial morphology calculated with the automated Harmony software. **(C)** qRT-PCR analysis of AATF, fusion (MFN1, MFN2) and fission (FIS1, DNM1L, MFF) genes performed on U138 and U87 cell lines transfected with siRNA targeting AATF (siAATF) or a control sequence (siControl). Data are presented as the mean ± SD of three independent experiments. *p≤0,05, **p≤0,01, ***p≤0,001, n.s. = not significant.

Supplementary Fig. S7 (related to Figure 5). AATF binds to the OXPHOS gene promoters and promotes their transcription through NRF-1. (A) qRT-PCR analysis of AATF and NRF-1 expression levels performed on U138 and U87 cell lines transfected with siRNA oligonucleotides targeting AATF (siAATF) or a control sequence (siControl). Data are presented as the mean ± SD of three independent experiments. *p≤0,05, n.s. = not significant. **(B)** Representative WB analysis of U138 cells transfected as in A and treated or not with MG132 20 µM for 24 hours. **(C)** Representative WB analysis with the indicated antibodies of cytoplasmic and nuclear fractions from equal amounts of U251 cells transfected as in A. **(D)** Nuclear extracts from U138 cells were subjected to immunoprecipitation with either AATF (left) or NRF-1 (right) antibodies. Immuno-precipitated complexes were then analysed by WB with the indicated antibodies. Input corresponds to 10% of the total extract used for immunoprecipitation.

Supplementary Fig. S8 (related to Figure 6). AATF is required for NRF-1 – dependent OXPHOS gene transcription. (A) Nuclear extracts from U251 cells were subjected to immunoprecipitation with a specific RNA polymerase II (phospho S5) antibody. Immuno-precipitated complexes were then analysed by WB with

the indicated antibodies. Input corresponds to 10% of the total extract used for immunoprecipitation. **(B)** Representative WB analysis with the indicated antibodies of U138 and U87 cells transfected with control siRNA (siControl) or two distinct siRNAs targeting AATF (siAATF #1 and siAATF #2).
