# Supplementary figures and images for: AATF supports proliferation of glioblastoma cells by sustaining mitochondrial respiration through an NRF-1-dependent mechanism
Source: Cell Death Dis. 2026 Mar 24;17(1):349. doi: 10.1038/s41419-026-08617-0 (PMC13039372; doi:10.1038/s41419-026-08617-0)

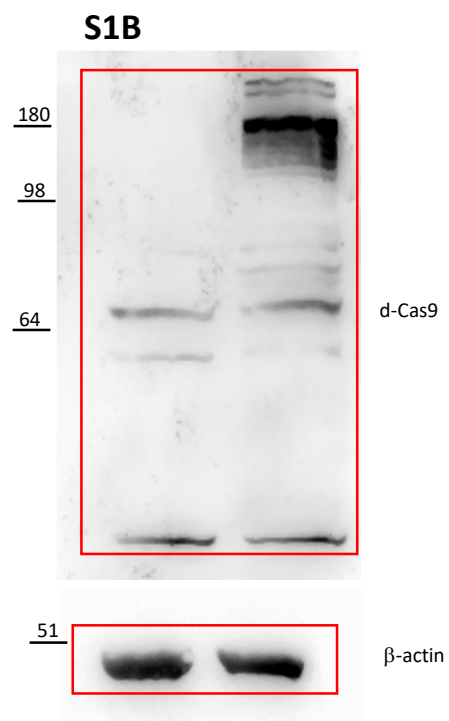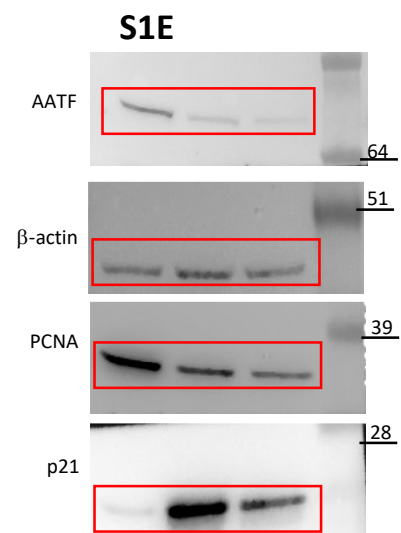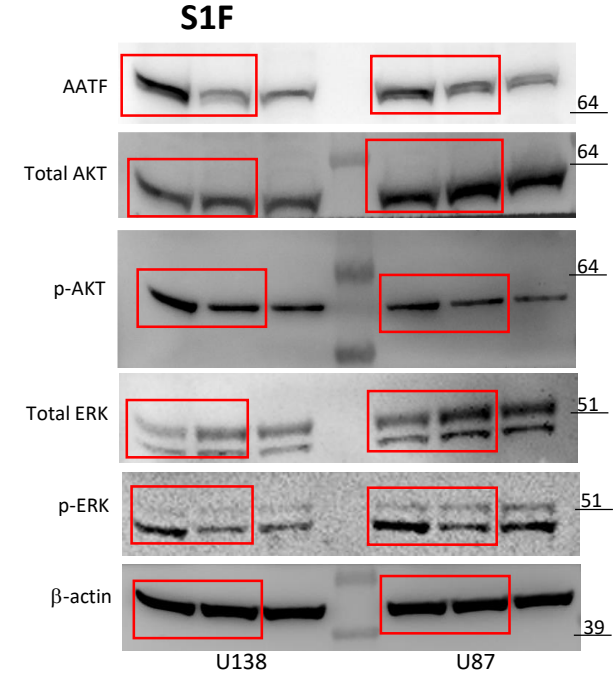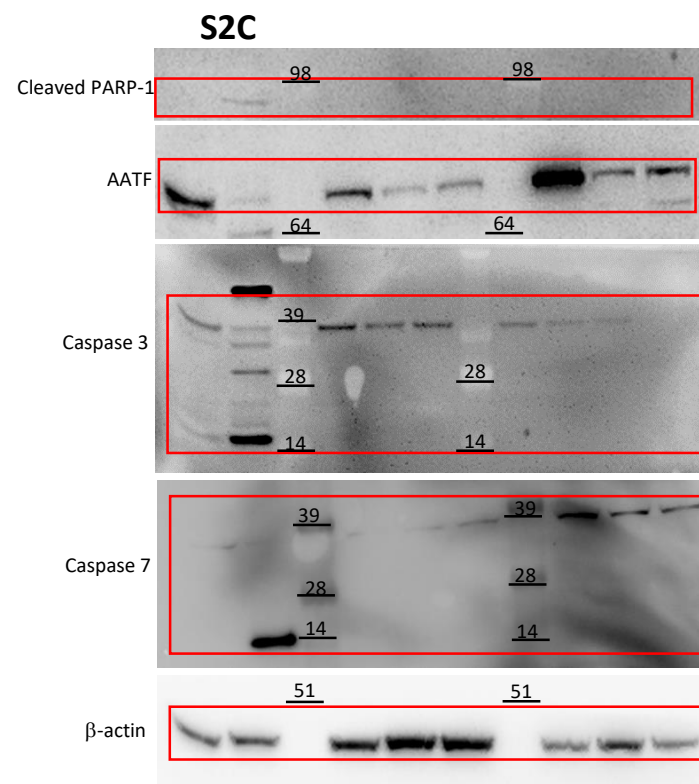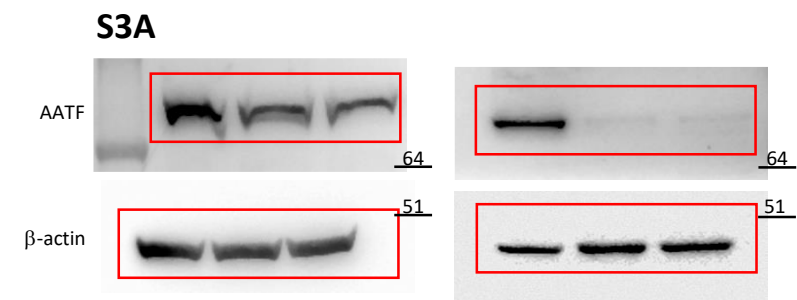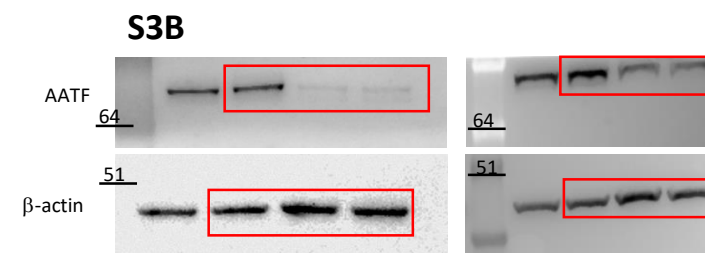

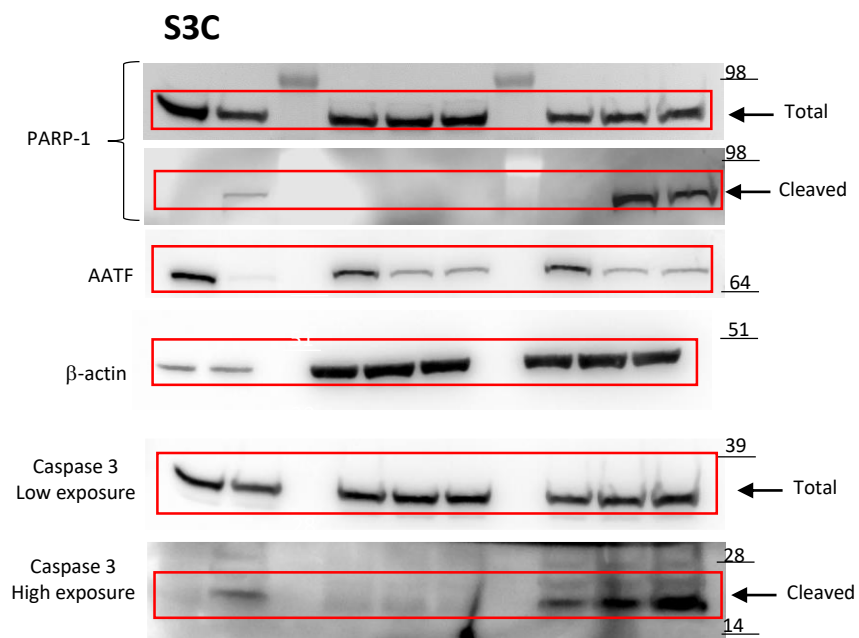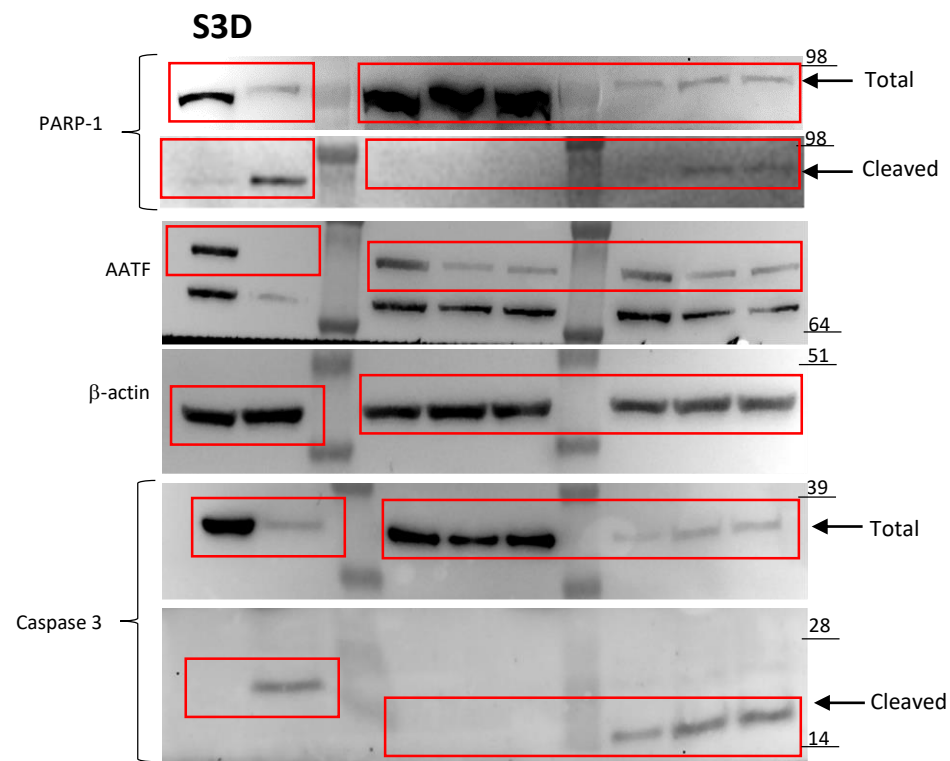

**S3D**

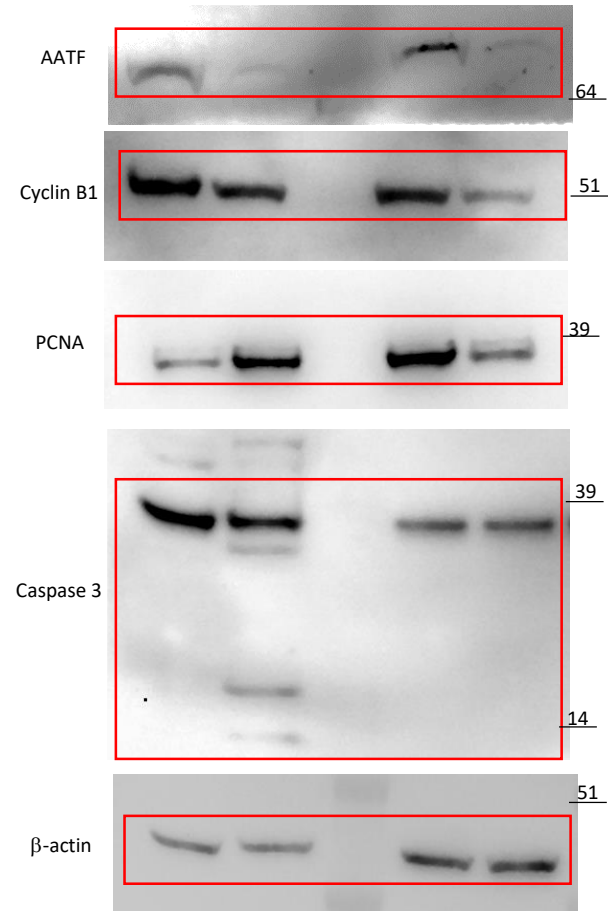

**S5E**

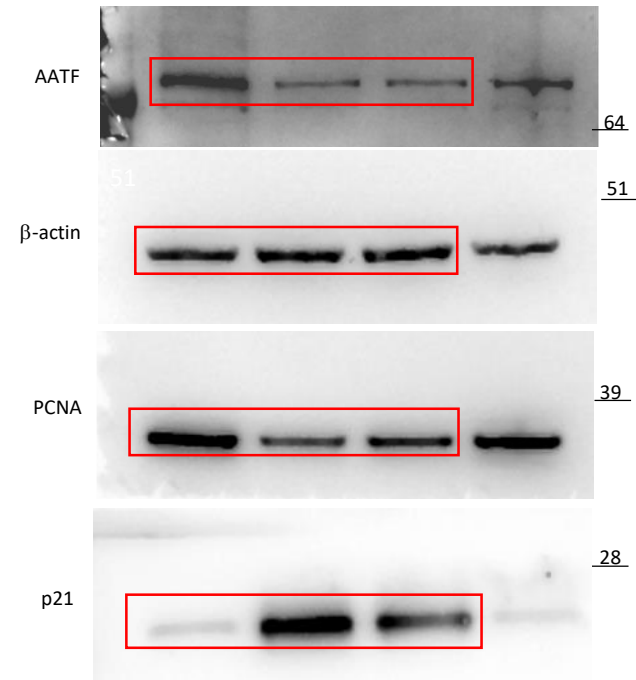

**5D**

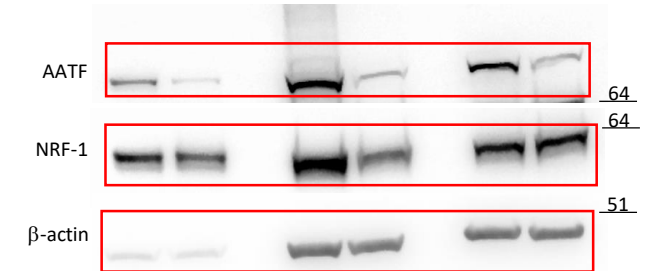

**5E**

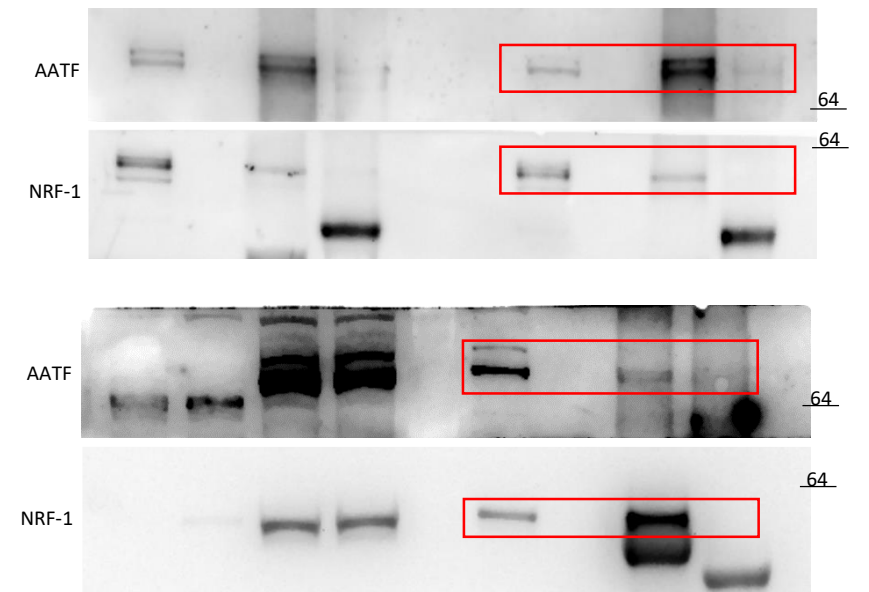

**S7B**

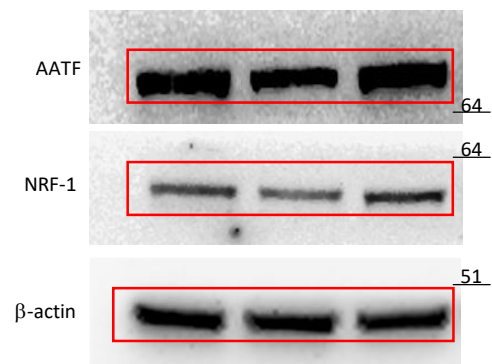

**S7C**

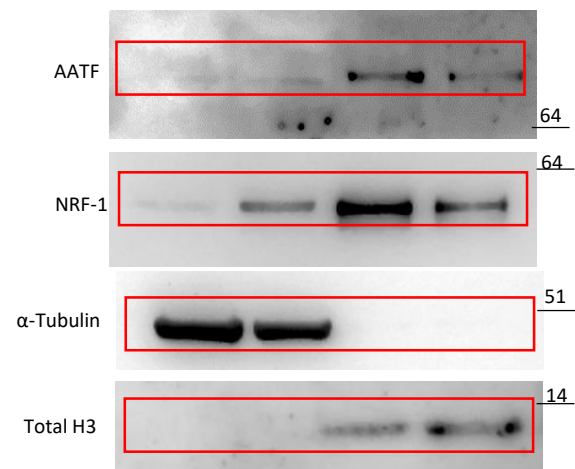

**S7E**

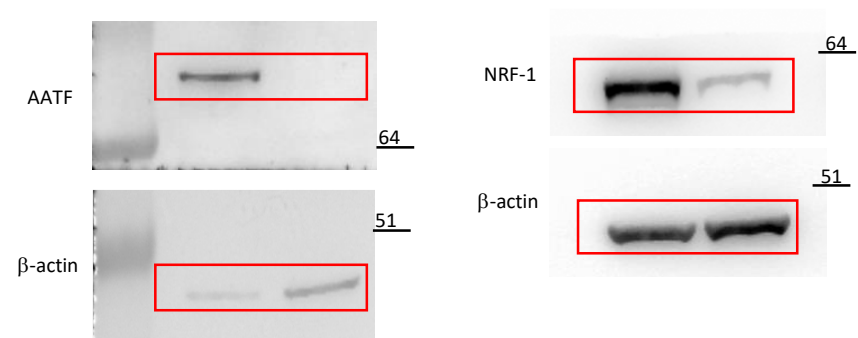

**S8A**

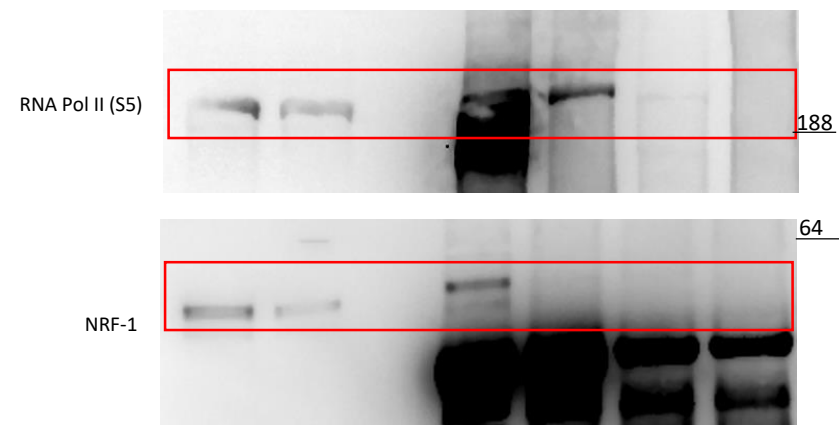

**S8B**

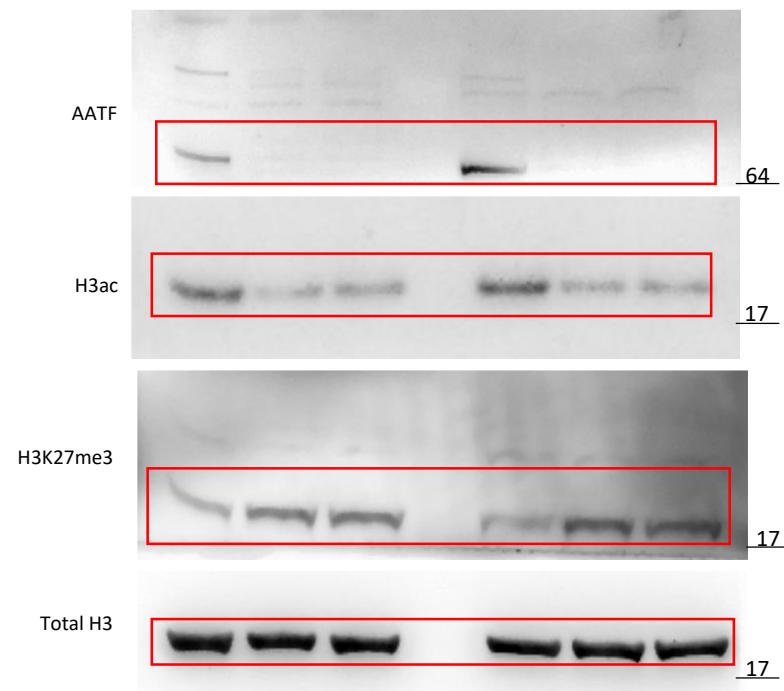

Supplement: Supplementary file 2 — Original western blots [file 41419_2026_8617_MOESM2_ESM.pdf]
